# Sulfated Steroid–Amino Acid Conjugates from the Irish Marine Sponge *Polymastia boletiformis*

**DOI:** 10.3390/md13041632

**Published:** 2015-03-24

**Authors:** Vangelis Smyrniotopoulos, Margaret Rae, Sylvia Soldatou, Yuanqing Ding, Carsten W. Wolff, Grace McCormack, Christina M. Coleman, Daneel Ferreira, Deniz Tasdemir

**Affiliations:** 1 School of Chemistry, National University of Ireland Galway, University Road, Galway, Ireland; E-Mails: evangelos.smyrniotopoulos@nuigalway.ie (V.S.); s.soldatou1@nuigalway.ie (S.S.); 2 Ryan Institute, National University of Ireland Galway, University Road, Galway, Ireland; E-Mail: margaret.rae@nuigalway.ie; 3 Marine Biodiscovery Laboratory, Marine Institute, Rinville, Oranmore, Co. Galway, Ireland; 4 Department of BioMolecular Sciences, Division of Pharmacognosy, and the National Center for Natural Products Research, School of Pharmacy, The University of Mississippi, University, MS 38677, USA; E-Mails: yqding@olemiss.edu (Y.D.); cmcoleman4321@gmail.com (C.M.C.); dferreir@olemiss.edu (D.F.); 5 Zoology, Ryan Institute, School of Natural Sciences, National University of Ireland Galway, University Road, Galway, Ireland; E-Mails: carstenwolff@hotmail.com (C.W.W.); grace.mccormack@nuigalway.ie (G.M.)

**Keywords:** marine, sponge, *Polymastia boletiformis*, sulfated steroid, antifungal activity

## Abstract

Antifungal bioactivity-guided fractionation of the organic extract of the sponge *Polymastia boletiformis*, collected from the west coast of Ireland, led to the isolation of two new sulfated steroid-amino acid conjugates (**1** and **2**). Extensive 1D and 2D NMR analyses in combination with quantum mechanical calculations of the electronic circular dichroism (ECD) spectra, optical rotation, and ^13^C chemical shifts were used to establish the chemical structures of **1** and **2**. Both compounds exhibited moderate antifungal activity against *Cladosporium cucumerinum*, while compound **2** was also active against *Candida albicans*. Marine natural products containing steroidal and amino acid constituents are extremely rare in nature.

## 1. Introduction

Marine sponges (Porifera) are primitive filter-feeders, yet they represent the richest and best studied sources of novel marine bioactive natural products [1]. Furthermore, sponges are considered to be producers of the highest sterol diversity amongst all animal phyla, presenting molecules with unique functionalization and structures, many with no terrestrial analogues [1,2,3]. The role of sterols in sponges is primarily functional, with sterols as constituents of cell membranes, and a secondary role as metabolic precursors for the production of diverse steroid classes [4,5,6]. Highly functionalized steroids are a growing group of metabolites that exhibit interesting biological and pharmacological properties, including ichthyotoxic, antihistaminic, cytotoxic, and antiviral activities [4,5].

*Polymastia boletiformis* (Lamarck, 1814, family Polymastiidae, order Hadromerida), is a brightly coloured orange-yellow fistulose sponge that grows on upper rock faces in the sublittoral zone and is widespread on the coasts of Britain and Ireland. Although not extensively studied, the genus *Polymastia* has been a source of various new fatty acids [7], carotenoids [8], and steroid compounds [9,10], including the antibiotic polymastiamides A–F [11,12]. Polymastiamides are the first examples of natural steroids with a side chain containing an amide bond linking the steroid part to a non-proteinaceous amino acid.

There has been a considerable increase in the frequency of fungal infections during the past decades. *Cladosporium cucumerinum* has been known as an important phytopathogen that causes scab disease in many commercial vegetables, resulting in significant losses [13]. *Candida albicans*, one of the most commonly encountered human pathogens, causes a variety of difficult-to-cure mucosal, skin, and systemic infections [14]. The declining number and efficacy of existing antifungal agents, the increased occurrence of systemic fungal infections, and rapid emergence of drug resistance underline the necessity for the discovery of new antifungal drug leads.

**Figure 1:**
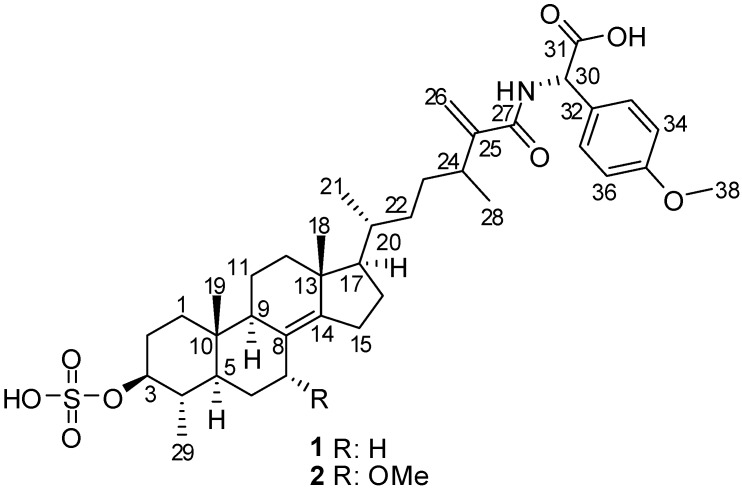
Structures of compounds **1** and **2**.

In the course of our ongoing investigations within the Beaufort Marine Biodiscovery Research program aimed at identification of novel bioactive metabolites from Irish marine resources [15], we have undertaken a chemical study of the Irish marine sponge *P. boletiformis*, as it displayed antifungal activity in initial screening studies. This report describes the bioactivity-guided isolation and structure elucidation of two new, minor sulfated steroid-amino acid conjugates, **1**, and its 7-methoxy derivative, **2** (Figure 1). Both compounds feature linkage of the non-proteinaceous α-amino acid *p*-methoxyphenylglycine with 4,24-dimethyl-3-*O*-sulfocholest-8(14),25(26)-dien-27-oic acid, via an amide bond. The molecular structures of the natural products were established on the basis of 1D and 2D NMR, IR, UV, HRMS, and experimental and calculated ECD, optical rotation, and ^13^C chemical shift data. The isolated metabolites were evaluated for their antifungal activity and were found to exhibit moderate antifungal activity against *C. cucumerinum*, while compound **2** was also active against *C. albicans*.

## 2. Results and Discussion

*P. boletiformis* specimens were collected by SCUBA from Roskeeda, Co. Galway (Ireland) and the CH_2_Cl_2_/MeOH extract of the freeze-dried sponge was subjected to a modified Kupchan partitioning scheme to yield *n*-hexane, CHCl_3_, and aqueous MeOH subextracts. Bioassay-guided separation of the antifungal aqueous MeOH-soluble fraction, which included a combination of C_18_ reversed-phase flash column chromatography (RP-FCC), Sephadex LH-20 size-exclusion chromatography, and repeated C_18_ reversed-phase (RP) HPLC purifications, yielded compounds **1** and **2**.

**Figure 2:**
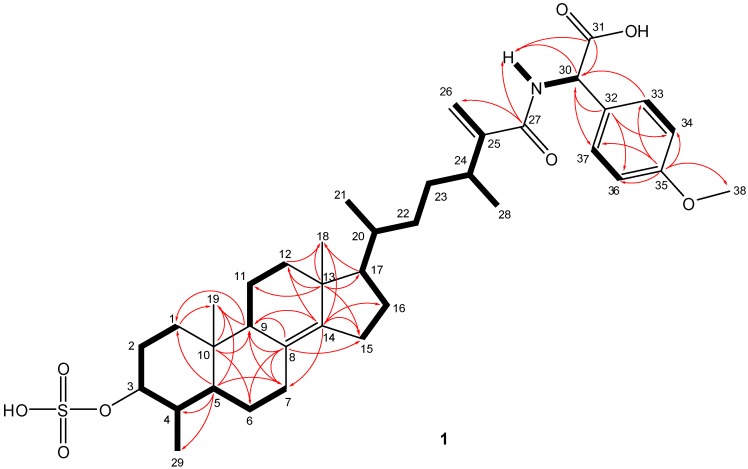
Key HMBC (arrows) and COSY (bold line) correlations observed in **1**.

Metabolite **1** was obtained as a white amorphous powder with ${\left[\text{α}\right]}_{\text{D}}^{20}\;$ +45.9 (*c* 0.03, MeOH). NMR data combined with the [M − H]^−^ ion at *m/z* 684.3570 in the HRESIMS of **1** suggested a molecular formula of C_38_H_55_NO_8_S (calcd for C_38_H_54_NO_8_S, 684.3576; Δ 0.75 ppm) requiring twelve indices of hydrogen deficiency. The structural characterization of compound **1** was established on the basis of extensive 1D and 2D NMR studies of data acquired in both DMSO-*d*_6_ and methanol-*d*_4_ (Table 1 and Supplementary Table S1). In DMSO-*d*_6_, both ^1^H and ^13^C-NMR spectra showed resonances typical of a steroid, displaying shielded resonances for two tertiary methyl groups (δ_H_ 0.64, δ_C_ 13.7; δ_H_ 0.78, δ_C_ 18.1) and three secondary methyls (δ_H_ 0.86, δ_C_ 19.0; δ_H_ 0.87, δ_C_ 15.5; δ_H_ 0.99, δ_C_ 19.7). Additional resonances accounting for one oxygenated methine (δ_H_ 3.53, δ_C_ 80.1), a tetrasubstituted double bond (δ_C_ 125.9, 141.4), a terminal olefinic methylene (δ_H_ 5.53 and 5.17, δ_C_ 113.9 and δ_C_ 151.4), a *p*-disubstituted benzene moiety (δ_H_ 6.74, δ_C_ 112.7; δ_H_ 7.18, δ_C_ 127.5; δ_C_ 134.9; δ_C_ 157.5), two carbonyl groups (δ_C_ 166.7 and δ_C_ 170.4), and one aromatic methoxy functionality (δ_H_ 3.67, δ_C_ 55.0) suggested that **1** was a steroid with an atypical, modified side chain. These functional groups also accounted for eight indices of hydrogen deficiency, with the remaining four assembling a typical four-ring steroid core. Furthermore, ^1^H and ^13^C-NMR data of **1** (Table 1) suggested the presence of a sulfate group based on deshielded resonances of the H-3/C-3 oxygenated methine (δ_H_ 3.53, δ_C_ 80.1) relative to a typical hydroxylated methine [16,17]. This was also supported by strong absorptions at 1214 and 1055 cm^−1^, characteristic of a sulfate functional group, present in the IR spectrum. An amide group was also evident due to the appearance of the resonance for a labile NH (δ_H_ 7.78) function in DMSO-*d*_6_, in addition to the broad IR absorption band centred at 3416 cm^−1^ (OH, NH stretch), and the intense bands observed at 1591 and 1548 cm^−1^. Analysis of the COSY and HMBC data led to the assignment of the ABCD steroid ring system in **1** (Figure 2). The COSY cross peaks between all neighbouring protons from H_2_-1 to H-7 assisted the assignment of rings A and B. HMBC correlations of angular methyl protons H_3_-19 with C-1, C-5, C-9, and C-10; of H-6α, Η-7β, and H-9 with C-8; of H-7β with C-9, along with the cross peaks of H-6β and H-9 with C-10; and of H-1β, H-4, Η-6β, H-7β, and H_3_-29 with C-5, validated the structure of the decalin AB rings. From the COSY spectrum it was possible to differentiate two discrete spin systems in the C and D rings; H-9/H_2_-11/H_2_-12 and H_2_-15/H_2_-16. HMBC correlations from the H_3_-18 methyl singlet to C-12, C-13, C-14, and C-17 and from C-13 to H-11β, Η-15β, Η-16α, and H-17 established the connectivity of C-12 and C-17 through the quaternary C-13. Diagnostic HMBC cross peaks between the olefinic C-14 and H-7α, H-9, H-12β, H_2_-15, and H_2_-16, along with correlations between C-8 and H-9 and H-15α assembled the ABCD steroidal rings. The COSY spectrum revealed useful information concerning the side chain. COSY correlations among all adjacent protons from C-17 to C-24, along with the long range coupling of methine H-24 with the sp^2^ methylene H-26b established the connection of spin system (i) C*H*17-C*H*20(C*H_3_*21)-C*H_2_*22-C*H_2_*23-C*H*24(C*H_3_*28)-C25(C*H_2_*26) of the side chain. Two more spin systems were evident within the side chain: (ii) N*H*-C*H*30; and (iii) C*H*33/37-C*H*34/36. Fragments (i) and (ii) could be connected on the basis of the HMBC correlations between C-27 and both terminal olefinic methylenes H_2_-26, as well as those between the amide proton NH and C-27, C-30, and C-31. The cross peaks in the HMBC spectrum between H-30 and C-31 clearly established the position of the carboxylic acid group at C-30. Complementary HMBC correlations between C-30 and H-33/37 and between H-30 and the aromatic carbons C-32 and C-33/37 validated the attachment of the *p*-disubstituted benzene moiety at the end of the side chain, thus connecting fragments (i), (ii), and (iii). Long-range heteronuclear couplings between H_3_-38 and C-35 secured the position of the methoxy group in the benzene ring and further established the gross structure for metabolite **1**. The amide linkage connecting the steroid side chain and d-cysteinolic acid in carolisterols A–C from the starfish *Styracaster caroli* is another rare example of marine natural products containing a steroidal and an amino acid component [18].

**Table 1:** ^1^H (600 MHz) and ^13^C (150 MHz) NMR chemical shifts of compound **1** and polymastiamide A (polyA) ^7a^ in DMSO-*d*_6_, δ in ppm, *J* values in Hz.

No.	^1^H (δ) m (*J*)			^13^C (δ) m		
	1	polyA	δ_Η_(1) − δ_Η_(polyA)	1	polyA	δ_C_(1) − δ_C_(polyA)
1	1.59 m 1.03 m	1.60 m 1.03 m	–0.01 -	35.8 t	35.8 t	-
2	2.10 m 1.28 m	2.10 m 1.28 m	- -	28.2 t	28.1 t	+0.1
3	3.53 ddd 10.9, 10.9, 4.4	3.53 m	-	80.1 d	80.1 d	-
4	1.21 m	1.21 m	-	37.2 d	37.1 d	+0.1
5	0.87 m	0.89 m	−0.02	50.7 d	50.7 d	-
6	1.65 m 0.90 m	1.64 m 0.87 m	+0.01 +0.03	24.7 t	24.7 t	-
7	2.32 br. dd 12.6, 3.7 1.65 d	2.32 m 1.63 m	- +0.02	29.2 t	29.2 t	-
8	-	-	-	125.9 s	125.8 s	+0.1
9	1.61 m	1.60 m	+0.01	48.7 d	48.7 d	-
10	-	-		36.9 s	36.8 s	+0.1
11	1.55 m 1.41 m	1.55 m 1.40 m	- +0.01	19.5 t	19.5 t	-
12	1.87 ddd 12.2, 3.2, 3.2 1.04 m	1.85 m 1.85 m	+0.02 −0.81	36.9 t	36.9 t	-
13	-	-	-	42.2 s	42.1 s	+0.1
14	-	-	-	141.4 s	141.4 s	-
15	2.18 br. dd 16.7, 10.2 2.11 m	2.16 m 2.08 m	+0.02 +0.03	25.3 t	25.3 t	-
16	1.71 dddd 13.1, 9.6, 7.3, 2.3 1.28 m	1.69 m 1.25 m	+0.02 +0.03	26.6 t	26.5 t	+0.1
17	1.02 m	0.97 m	+0.05	56.3 d	56.3 d	-
18	0.78 s	0.76 s	+0.02	18.1 q	18.0 q	+0.1
19	0.64 s	0.63 s	+0.01	13.7 q	13.6 q	+0.1
20	1.37 m	1.34 m	+0.03	34.1 d	33.9 d	+0.2
21	0.86 d 6.6	0.83 d 6.5	+0.03	19.0 q	18.9 q	+0.1
22	1.33 m 0.98 m	1.32 m 0.96 m	+0.01 +0.02	32.5 t	32.5 t	-
23	1.48 dddd 12.4, 12.0, 6.7, 4.3 1.15 dddd 12.0, 10.6, 6.6, 4.8	1.46 m 1.13 m	+0.02 +0.02	31.3 t	31.3 t	-
24	2.50 m	2.54 m	+0.04	35.0 d	34.9 d	+0.1
25	-	-	-	151.4 s	149.5 s	+1.9
26	5.53 s 5.17 s	5.61 s 5.21 s	−0.08 −0.04	113.9 t	115.8 t	−1.9
27	-	-	-	166.7 s	168.5 s	−1.8
28	0.99 d 6.9	0.98 d 6.8	+0.01	19.7 q	19.6 q	+0.1
29	0.87 d 6.3	0.87 d 6.2	-	15.5 q	15.5 q	-
30	4.60 d 5.8	5.35 d 7.6	−0.75	58.2 d	55.6 d	+2.6
31	-	-	-	170.4 s	172.2 s	−1.8
32	-	-	-	134.9 s	129.3 s	+5.6
33	7.18 d 8.6	7.31 d 8.7	−0.13	127.5 d	129.1 d	−1.6
34	6.74 d 8.6	6.88 d 8.7	−0.14	112.7 d	113.6 d	−0.9
35	-	-	-	157.5 s	158.8 s	−1.3
36	6.74 d 8.6	6.88 d 8.7	−0.14	112.7 d	113.6 d	−0.9
37	7.18 d 8.6	7.31 d 8.7	−0.13	127.5 d	129.1 d	−1.6
38	3.67 s	3.72 s	−0.05	55.0 q	55.1 q	−0.1
N*H*	7.78 d 5.8	8.48 d 7.5	−0.70	-	-	

**Figure 3:**
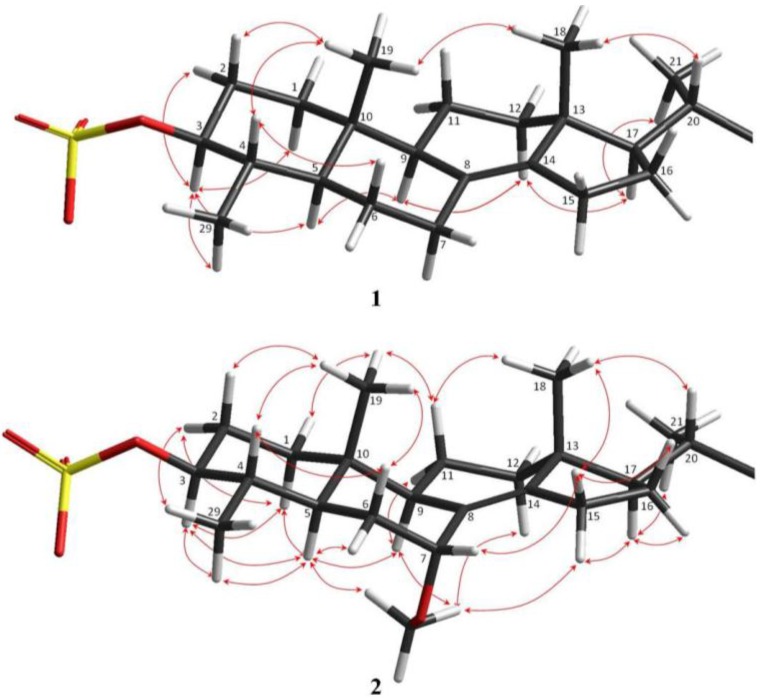
Relative configurations and key NOESY correlations observed within the steroid portion of **1** and **2**.

The relative configurations of the steroidal ring stereogenic centres of **1** were deduced from NOE studies and *J* values in the ^1^H NMR spectra. The NOESY data indicated that **1** contained the common 5α/10β/9α/13β steroid nucleus (Figure 3). Key NOE correlations of angular methyl protons H_3_-19 with Η-4, as well as cross peaks of H-3 with Η-5 and H_3_-29, and of H-5 with H-9 established the *trans*-fusion of the AB decalin ring system, indicating that H-4 and H_3_-19 were cofacial and β-oriented, while H_3_-29, H-3, H-5, and H-9 were cofacially α-oriented. H-3 was designated axial based on a large vicinal coupling constant (*J* = 10.9 Hz), and the NOE observed between H-3 and H-5. The NOE correlations from Η_3_-18 to both H_3_-19 and H-20, and an additional cross peak between H-17 and H-21 established the relative configurations of the remaining stereocentres of rings C and D, and the β-orientation of the side chain. However, the NOESY data did not allow definition of the relative configurations of the C-24 and C-30 stereogenic centres of the freely rotating side chain.

Comparison of the gross NMR data of **1** with those reported for polymastiamide A obtained from a *P. boletiformis* specimen from Norway by Kong and Anderson in 1993 [11] indicated similar planar structures. The NMR data in DMSO-*d*_6_ (Table 1) displayed similar ^1^H and ^13^C chemical shifts, for all carbons and protons in the rigid steroid portion (ABCD rings). Notable differences were observed in the chemical shifts of the carbons and protons in the proximity of the chiral environment of C-24 (C-24 to C-27) and C-30 (especially C-30 to C-32). Both ^1^H and ^13^C-NMR chemical shift values of **1** were solvent dependent (Table 1, Supplementary Table S1). For example, in methanol-*d*_4_, H-30 appeared at δ_H_ 5.25 in **1**, comparable to that of polymastiamide A (δ_H_ 5.35) in DMSO-*d*_6_. The above-mentioned data pointed out the necessity for a detailed investigation of the absolute configuration at stereocentres C-24 and C-30.

**Figure 4:**
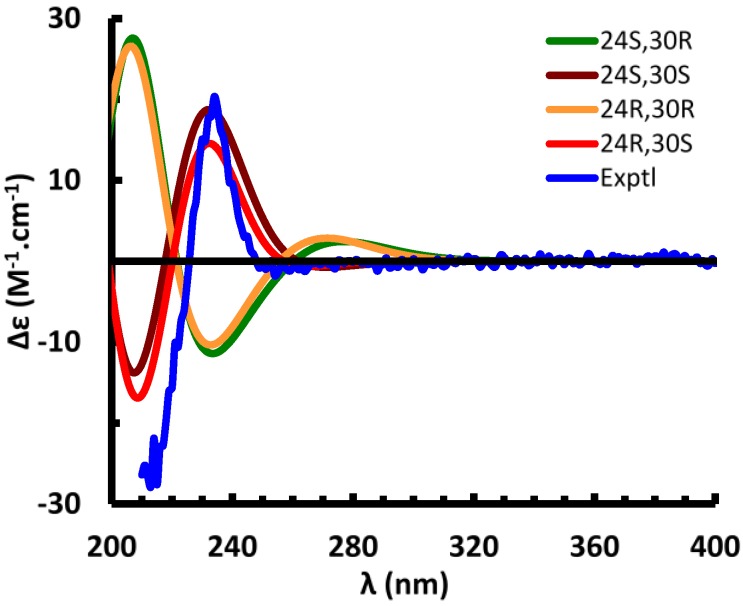
Experimental and calculated electronic circular dichroism (ECD) spectra of compound **1**.

Suitable crystals of **1** could not be obtained. The absolute configuration of the side chain was therefore investigated by comparing the experimental electronic circular dichroism (ECD) spectrum of **1** to the ECD spectra predicted from quantum mechanical calculations for the four possible diastereomers. The ECD spectra [19,20,21,22] were simulated to assign the absolute configuration (AC) of **1** by using the Gaussian 09 software packages [23]. The configurations of the 4-methoxyphenylglycine moiety and C-24 were assigned as *S*- or *R*-, denoted as (24*R*,30*R*)-, (24*R*,30*S*)-, (24*S*,30*R*)-, and (24*S*,30*S*)-**1**, respectively. The OPLS-2005 force field in MacroModel [24] was employed to perform the conformational random search with an energy window of 130 kJ/mol, and yielded a total of 591 conformers for (24*S*,30*R*)-**1** and 546 conformers for (24*S*,30*S*)-**1** (Supplementary Figure S1). Fourteen of the (24*S*,30*S*)-**1** (energy cut-off, 20 kJ/mol) and 44 of the (24*S*,30*R*)-**1** conformers (energy cut-off, 15 kJ/mol) were included for the geometric optimization, followed by harmonic vibrational frequency computation to confirm these as energy minima. Nine of the (24*S*,30*S*)-**1** conformers were relocated at the B3LYP/6-31G** level in the gas phase, among which conformer 7 (Supplementary Table S3 and Figure S2) was calculated to dominantly occupy 97.5% of the conformational itinerary. The ECD spectrum of the predominant conformer was simulated at the B3LYP/6-31G** level in the gas phase and matched very well with the experimental spectrum (Figure 4). Conformer 1 of (24*S*,30*R*)**-1** occupies 96.7% of the conformational itinerary and its computed ECD spectrum was opposite to the experimental spectrum (Figure 4). Optimized geometries of the predominant conformers of (24*S*,30*S*)- and (24*S*,30*R*)-**1** show strong intramolecular hydrogen bonding between the hydroxy group of the carboxylic moiety and the C-3 sulfate group. The distance of (C3-O-SO_2_-)O…H(-O-C31) was optimized as 1.519 and 1.469 Å for (24*S*,30*S*)- and (24*S*,30*R*)**-1**, respectively (Supplementary Figure S2). The C25-C27(O)-N-C30-C31(O)-O-H…O-S-O “backbone” in (24*S*,30*S*)-**1** is coplanar, thus delocalized (π_1_), with the delocalized 4-methoxyphenyl group (π_2_) perpendicular to the plane. Molecular orbital analysis of (24*S*,30*S*)-**1** indicated the experimentally observed positive Cotton effect (CE) at 234 nm corresponds to the electronic transitions at 230.9 and 230.2 nm (Supplementary Table S4) from Orb182 (π_C-8=C-14_, Supplementary Figure S3) to Orb187 (π*_2_), and from Orb183 (π_2_) to Orb188 (π*_2_). The negative CE at 213 nm results predominantly from the transition at 201 nm from Orb182 (π_C-8=C-14_) to Orb189 (π*_C-8=C-14_), and the transitions at 216 and 213 nm from Orb180 to Orb187, and from Orb181 to Orb188. The similar CEs result from the electronic transitions between π_1_, π_2_, π_C-8=C-14_, and the unoccupied orbitals, but did not permit definition of the absolute configuration at C-24. This is confirmed by the finding that the calculated ECD spectra of (24*R*,30*S*)- and (24*R*,30*R*)-**1** are similar to those of (24*S*,30*S*)- and (24*S*,30*R*)-**1**, respectively (Figure 4). An attempt to differentiate these pairs by calculation of their specific rotations was also unsuccessful. The calculated specific rotations of (24*S*,30*R*)- and (24*R*,30*R*)-**1** are −62.9 and −73.5 at the B3LYP/6-311++G**//B3LYP/6-31G** level in the gas phase, and those of (24*R*,30*S*)- and (24*S*,30*S*)-**1** are 96.4 and 96.2, respectively [25].

The comparison of quantum chemical calculated ^13^C NMR chemical shifts with the experimental data was considered as a useful tool to assist the assignment of absolute configuration of chiral molecules [26]. However, due to the large size of compound **1**, the computation of ^13^C NMR shielding constants of the above conformers failed when using the GIAO technique at the mPW1PW91-SCRF (PCM, MeOH)/6-311++G** level of theory [27]. The calculated ^13^C NMR chemical shifts of (24*R*,30*S*)**-1** and (24*S*,30*S*)**-1** at the B3LYP/6-31G** level in the gas phase show very high similarity, differing less than 2.4 ppm and roughly match the experimental data (Supplementary Table S5). Even though the calculated chemical shifts of C-28 differ by 4.7 ppm, both match the experimental value. The noticeable difference is that the calculated chemical shifts of C-24 differ by 5.3 ppm, whereas that in (24*S*,30*S*)**-1** was calculated as 43.1 ppm that was closer to the experimental value of 35.0 ppm than that in (24*R*,30*S*)**-1** which was computed as 48.4 ppm, implying that most likely the absolute configuration at C-24 is (24*S*).

In summary, the ECD study provided unambiguous confirmation of the (30*S*) absolute configuration, but could not differentiate between (24*R*) and (24*S*) configurations. Calculation of the specific rotations of the four diastereomers similarly failed to differentiate the (24*R*) and (24*S*) configurations. The (30*S*)-configuration matches with the reported assignment for polymastiamide A that resulted from hydrolysis of the amino acid residue (*p*-hydroxyphenylglycine) from the desulfated methyl ester of the compound by Marfey’s method [11]. The l-configuration of the amino acid residue in polymastiamide A dictated (30*S*) configuration at C-30 while the configuration of C-24 remained unidentified [11]. Compound **1** and polymastiamide A have similar specific rotations (${\left[\text{α}\right]}_{\text{D}}^{20}\;$ +45.9 for **1**, (${\left[\text{α}\right]}_{\text{D}}^{20}\;$ +67.4 for polymastiamide A), both acquired in MeOH. Based on these data we conclude that **1** and polymastiamide A differ at the C-24 stereogenic centre; however, the C-24 absolute configuration remains undefined in both compounds.

The molecular formula for the optically active (${\left[\text{α}\right]}_{\text{D}}^{20}\;$+33.8, *c* 0.04, MeOH) metabolite **2**, C_39_H_57_NO_9_S, was derived from NMR data and the HRESIMS ion at *m/z* 714.3674 ([M − H]^−^, calcd for C_39_H_56_NO_9_S, 714.3681; Δ 1.07 ppm). Comparison of the molecular formula of **2** with that of **1** revealed that **2** contained a methoxymethine rather than a methylene functionality. The ^1^H and ^13^C-NMR resonances in both DMSO-*d*_6_ and methanol-*d*_4_, fully assigned through COSY, HSQC, and HMBC experiments (Table 2 and Supplementary Table S2) indicated that the only difference between the two molecules was the presence of a C-7 methoxy group in **2** (δ_H_ 3.94, δ_C_ 73.3). The COSY couplings from H-7 to both H-6α and H-6β, and the HMBC correlations from H-7 to C-5, C-6, C-9 and C-14, and from C-7 to H-6α, H-9 and C7-OMe confirmed the position of the methoxy group at C-7. Once the planar structure of **2** was defined, its relative configuration was established on the basis of 2D NMR NOESY to be the same as that of **1** (Figure 3). The NOESY correlations between H-7 and H-6β, between H-6β and H_3_-19, and between C7-OCH_3_ and H-5 and H-9 confirmed the α-axial orientation of the methoxy group. The similarity of the experimental ECD spectra of compounds **1** and **2** indicates they have the same absolute configurations for all stereogenic centres, with the exception of the (7*R*)-configuration of **2**.

**Table 2:** ^1^H (600 MHz) and ^13^C (150 MHz) NMR chemical shifts of compound **2** in DMSO-*d*_6_, δ in ppm, *J* values in Hz.

No.	^1^H (δ) m (*J*)	^13^C (δ) m
1	1.56 m 1.02 m	35.6 t
2	2.09 dm 12.2 1.28 m	28.1 t
3	3.50 ddd 11.2, 10.0, 4.8	80.2 d
4	1.20 m	36.8 d
5	1.22 m	44.1 d
6	1.83 m 1.07 m	30.4 t
7	3.94 br. s	73.3 d
8	-	124.5 s
9	1.87 m	43.4 d
10	-	37.1 s
11	1.56 m 1.36 m	19.8 t
12	1.89 m 1.04 m	36.6 t
13	-	42.8 s
14	-	147.9 s
15	2.37 ddd 17.6, 9.5, 8.7 2.21 br. dd 17.6, 12.4	25.0 t
16	1.76 m 1.31 m	26.4 t
17	1.05 m	56.6 d
18	0.80 s	17.4 q
19	0.63 s	12.9 q
20	1.38 m	34.1 d
21	0.89 d 6.4	19.0 q
22	1.36 m 0.97 m	32.5 t
23	1.49 m 1.17 m	31.5 t
24	2.50 m	35.0 d
25	-	151.3 s
26	5.53 s 5.17 br. s	114.0 t
27	-	166.7 s
28	1.00 d 6.9	19.0 q
29	0.84 d 5.8	15.4 q
30	4.57 d 5.0	58.2 d
31	-	170.5 s
32	-	134.9 s
33	7.18 d 8.4	127.5 d
34	6.74 d 8.4	112.7 d
35	-	157.5 s
36	6.74 d 8.4	112.7 d
37	7.18 d 8.4	127.5 d
38	3.69 s	55.0 q
39	3.03 s	53.5 q
NH	7.78 d 5.0	-

Compounds **1** and **2** were evaluated for their antifungal activity against *Cladosporium cucumerinum* and *Candida albicans*, and for their antibacterial activity against *Staphylococcus aureus*. The assays demonstrated that **1** and **2** showed moderate inhibitory effects on *C. cucumerinum* at 60 and 30 μg/disc, respectively. The observed diameters of their inhibition zones were 8.0 and 10.1 mm, respectively. Compound **2** was also found to possess significant activity at 100 μg/disc against *C. albicans*, displaying an inhibition zone diameter of 9.8 mm. The diameters of inhibition zones of the positive controls used in the assays, CuSO_4_ and nystatin, were 16.8 and 25.8 mm, respectively. No significant activity was demonstrated against *S. aureus*. Polymastiamide A was reported by Kong *et al*., to be active against both human pathogens *S. aureus* and *C. albicans* [11].

## 3. Experimental Section

### 3.1. General Experimental Procedures

Optical rotations were measured at the sodium D line (589.3 nm), at 20 °C on a Unipol L1000 Schmidt + Haensch polarimeter with a 10 cm cell. UV spectra were acquired in spectroscopic grade MeOH or CHCl_3_ on a Varian, Cary 100 UV-Vis spectrophotometer. IR spectra were obtained using a Perkin Elmer 400 FT-IR spectrometer with ATR attached. NMR spectra were recorded using a Jeol 400 MHz, a Varian 500 MHz, or an Agilent 600 MHz spectrometer with a cryo probe. Chemical shifts are given on the δ (ppm) scale using TMS as internal standard, and *J* values in Hz. High resolution mass spectrometric data were acquired on an Agilent 1290 Infinity-QTOF 6540 UPLC/MS system, with electrospray ionization (ESI) in the negative ion mode. C_18_ silica gel (40–63 μm; Merck, Tullagreen, Ireland), and Sephadex LH-20 (GE Healthcare, Cork, Ireland) were used for column chromatography (CC). Thin layer chromatography (TLC) was performed with Kieselgel 60 F_254_ aluminium support plates (Merck, Tullagreen, Ireland) and spots were detected after spraying with 6% vanillin and 15% H_2_SO_4_ in MeOH reagents and charring. HPLC separations were conducted using an Agilent 1260 model equipped with a diode array detector, either on semi-preparative (250 × 10 mm, 5 μm) or on analytical (150 × 4.6 mm, 5 μm) Kaseisorb ODS2000 HPLC columns. The chromatography was monitored at 254 nm. All solvents were of HPLC grade and were purchased from Sigma Aldrich (Arklow, Ireland).

### 3.2. Biological Material

Specimens of *Polymastia boletiformis* were collected by SCUBA diving in Roskeeda, Co. Galway, Ireland, at a depth of 6–9 m in August 2009. A voucher specimen has been deposited at the Department of Zoology, Ryan Institute, School of Natural Sciences, National University of Ireland, Galway, under reference MIIG30041.

### 3.3. Extraction and Isolation

*Polymastia boletiformis* was initially freeze-dried (9.1 g dry weight) and then exhaustively extracted with CH_2_Cl_2_ (3 × 500 mL) and MeOH (3 × 500 mL) at room temperature. The combined extracts were concentrated under reduced pressure at 35 °C to give a dark brown residue (1.44 g) that was subjected to a modified Kupchan partition. Briefly, the crude extract was partitioned between 10% aqueous MeOH (500 mL) and *n*-hexane (3 × 500 mL). The water concentration was increased to 35%, before extracting three times with CHCl_3_ (3 × 500 mL). Evaporation of the solvent under reduced pressure at 35 °C afforded the *n*-hexane (0.33 g), CHCl_3_ (0.06 g), and aqueous MeOH (1.04 g) subextracts. The aqueous subextract (W) was fractionated by RP-FCC (Lichroprep RP18, 40–63 μm), eluting with gradient mixtures of water and MeOH of reducing polarity to afford nine fractions. Antifungal activity against *C. cucumerinum* was detected in fractions 6 (W6) and 7 (W7), eluted with 60 and 70% MeOH in water, respectively. Fraction W7 (7 mg) afforded compound **1** (1.0 mg) after repeated purifications with RP-HPLC performed on a 5 μm, 150 × 4.6 mm Kaseisorb ODS2000 column. A binary eluent consisting of H_2_O (solvent A) and MeCN (solvent B) delivered at 1.2 mL/min was used with the following gradient profile: 0 min, 20% B; 10 min, 40% B. Fraction W6 (50 mg) was chromatographed on a Sephadex LH-20 column eluted with MeOH to give a fraction containing steroid-like compounds (28.8 mg). Final purification of **2** (1.3 mg) was achieved by repeated semi-preparative RP-HPLC of the latter fraction on a 5 μm, 250 × 10 mm Kaseisorb ODS2000 column, using a gradient binary solvent system, consisting of H_2_O (solvent A) and MeCN (solvent B), at a flow rate of 1.25 mL/min: 0 min, 5% B; 10 min, 20% B; 55 min, 40% B.

Compound **1**. White amorphous powder; ${\left[\text{α}\right]}_{\text{D}}^{20}\;$ +45.9, (*c* 0.03, MeOH); UV ${\text{λ}}_{\text{max}}^{CH_{3}OH}$ (log ε): 287 (2.80); IR (film) ν_max_ 3416, 1591, 1548, 1214, 1055 and 1033 cm^−1^; NMR data (DMSO-*d*_6_, methanol-*d*_4_), see Table 1 and Supplementary Table S1; HRESIMS *m/z* 684.3570 [M − H]^−^, (calcd for C_38_H_54_NO_8_S, 684.3576; Δ 0.75 ppm).

Compound **2**. White amorphous powder; ${\left[\text{α}\right]}_{\text{D}}^{20}\;$ +33.8 (*c* 0.04, MeOH); UV ${\text{λ}}_{\text{max}}^{CH_{3}OH}$ nm (log ε): 289 (2.87), 239 (2.30); IR (film) ν_max_ cm^−1^ 3401, 1600, 1511, 1214, 1062 and 1033 cm^−1^; NMR data (DMSO-*d*_6_, CD_3_OD), see Table 2 and Supplementary Table S2; HRESIMS *m/z* 714.3674 [M − H]^−^, (calcd for C_39_H_56_NO_9_S, 714.3681; Δ 1.07 ppm).

### 3.4. Disc Diffusion Assay against C. cucumerinum

Freeze-dried ampoules of spores from the plant fungus *C. cucumerinum* (IMI 299104) were obtained from the Centre for Agriculture and Biosciences International UK and cultures of the fungus were maintained to harvest spores. These spores were subsequently inoculated onto potato dextrose agar petri dishes upon which antibiotic disks (6 mm) impregnated with MeOH solutions of the fractions or isolated compounds (100 μg/disc for fractions, and 30 and 60 μg/disc for pure compounds) were placed. Plates were incubated at 25 °C and observed at 24 and 48 h [28]. The positive control was CuSO_4_ (3000 μg on disk) and the negative control was the solvent, MeOH.

### 3.5. Disc Diffusion Assay against C. albicans and S. aureus

About 20 mL of sterilized nutrient agar medium was poured into each sterile petri plate and allowed to solidify. Inoculums of the test pathogens *C. albicans* (CBS 562 strain) and *S. aureus* (8325-4 MSSA strain) were prepared using a McFarland 0.5 standard and left at RT for 15 min. The inoculums were evenly spread over the appropriate media by using a sterile cotton swab and incubated for 15 min. Sterile discs (6 mm) impregnated with 30 μL (100 μg/disc) of the compounds dissolved in MeOH and the controls were placed on the surface of the media using sterile forceps. MeOH was used as the negative control, while nystatin (33.3 μg/disc) and ampicillin (10 μg/disc) were used as positive controls for *C. albicans* and *S. aureus*, respectively. Both *C. albicans* (incubated at 30 °C) and *S. aureus* (incubated at 37 °C) were observed at 24 h for development of inhibition zones.

## 4. Conclusions

In our continuing investigation aimed at the biological screening and isolation of bioactive metabolites from Irish marine organisms, two new sulfated steroid–amino acid conjugates, **1** and its 7-methoxy derivative **2**, were isolated from the Irish marine sponge *Polymastia boletiformis*. Marine natural products containing steroidal and amino acid components are rare and have previously been encountered only in polymastiamides from the same sponge collected from Norway [11,12], and in carolisterols from the starfish *Styracaster caroli* [18]. The discovery of **1** and **2** presents a unique case, as **1** was found to share the same planar structure with polymastiamide A [11], yet the two compounds differ significantly in both proton and carbon resonances of the side chain atoms, especially around the C-24 and C-30 stereogenic centres. ECD and optical rotation calculations have been applied to **1** and permitted definition of absolute configuration, except for at the C-24 stereogenic centre. Metabolites **1** and **2** exhibited moderate antifungal activity against the plant pathogen *C. cucumerinum*, while **2** was also found active against *C. albicans*. The latter activity was also reported for polymastiamide A [11].

## Supplementary Files

Supplementary File 1
