# Supplementary material for: Sulfated Steroid–Amino Acid Conjugates from the Irish Marine Sponge Polymastia boletiformis
Source: Mar Drugs. 2015 Mar 24;13(4):1632–46. doi: 10.3390/md13041632 (PMC4413178; doi:10.3390/md13041632)
Supplement: Supplementary File 1 [file marinedrugs-13-01632-s001.pdf]

Supplementary Information

Table of Contents

Table S1. ^{1}H (600 MHz) and ^{13}C (150 MHz) NMR chemical shifts (DMSO-d_6 and CD_3OD), NOESY and HMBC correlations of compound **1**.
Table S2. ^{1}H (600 MHz) and ^{13}C (150 MHz) NMR chemical shifts (DMSO-d_6 and CD_3OD), NOESY and HMBC correlations of compound **2**.
Table S3. Conformational Analysis of (24*S*,30*S*)-**1** at the B3LYP/6-31G** Level in the Gas Phase.
Table S4. Important Transition States, Related Rotatory Strengths, and Oscillator Strengths of (24*S*,30*S*)-**1** at the B3LYP/6-31G* Level in the Gas Phase.
Table S5. Experimental and calculated NMR chemical shifts of **1**.
Figure S1. Random conformational search of (24*S*,30*R*) and (24*S*,30*S*)-**1** with an energy window of 130 kJ/mol.
Figure S2. Optimized geometries of predominant conformers **1** at the B3LYP/6-31G** level in the gas phase.
Figure S3. Molecular orbitals involved in the key transitions in the calculated ECD spectrum of (24*S*,30*S*)-**1** at the B3LYP/6-31G** level in the gas phase.
Figure S4. ^{1}H NMR spectrum (600 MHz, DMSO-d_6) of compound **1**.
Figure S5. ^{13}C NMR spectrum (150 MHz, DMSO-d_6) of compound **1**.
Figure S6. ^{1}H NMR spectrum (600 MHz, CD_3OD) of compound **1**.
Figure S7. ^{13}C NMR spectrum (150 MHz, CD_3OD) of compound **1**.
Figure S8. ^{1}H-^{1}H COSY spectrum (DMSO-d_6) of compound **1**.
Figure S9. HSQC spectrum (DMSO-d_6) of compound **1**.
Figure S10. HMBC spectrum (DMSO-d_6) of compound **1**.
Figure S11. NOESY spectrum (DMSO-d_6) of compound **1**.
Figure S12. ^{1}H-^{1}H COSY spectrum (CD_3OD) of compound **1**.
Figure S13. HSQC spectrum (CD_3OD) of compound **1**.
Figure S14. ESIMS spectrum of compound **1**.
Figure S15. IR spectrum of compound **1**.
Figure S16. ^{1}H NMR spectrum (600 MHz, DMSO-d_6) of compound **2**.
Figure S17. ^{13}C NMR spectrum (150 MHz, DMSO-d_6) of compound **2**.
Figure S18. ^{1}H NMR spectrum (600 MHz, CD_3OD) of compound **2**.
Figure S19. ^{13}C NMR spectrum (150 MHz, CD_3OD) of compound **2**.
Figure S20. ^{1}H-^{1}H COSY spectrum (CD_3OD) of compound **2**.
Figure S21. HSQC spectrum (CD_3OD) of compound **2**.
Figure S22. HMBC spectrum (CD_3OD) of compound **2**.
Figure S23. NOESY spectrum (CD_3OD) of compound **2**.
Figure S24. HSQC spectrum (DMSO-d_6) of compound **2**.
Figure S25. ESIMS spectrum of compound **2**.
Figure S26. IR spectrum of compound **2**.

Table S1. ^{1}H (600 MHz) and ^{13}C (150 MHz) NMR chemical shifts (DMSO-d_6 and CD_3OD), NOESY and HMBC correlations of compound **1**.

No.	DMSO-d_6		CD_3OD		NOESY	DMSO-d_6	CD_3OD	Type	HMBC
	^{1}H (δ)	m (*J*)	^{1}H (δ)	m (*J*)		^{13}C (δ)	^{13}C (δ)		(^{13}C→^{1}H)
1	β 1.59<br>α 1.03	m<br>m	β 1.73<br>α 1.18	m<br>m	11β<br>2α, 3	35.8	37.4	CH_2	19
2	α 2.10<br>β 1.28	m<br>m	α 2.26<br>β 1.56	m<br>m	1α, 1β, 3<br>19	28.2	29.4	CH_2	
3	3.53	ddd 10.9, 10.9, 4.4	3.86	ddd 11.0, 10.6, 4.8	1α, 2α, 5, 29	80.1	85.2	CH	4, 29
4	1.21	m	1.44	m	6β, 19	37.2	38.9	CH	29
5	0.87	m	0.97	m	3, 9	50.7	52.6	CH	1β, 4, 6β, 7β, 19, 29
6	α 1.65<br>β 0.90	m<br>m	α 1.77<br>β 1.02	m<br>m	 4, 7β	24.7	26.2	CH_2	5, 7α
7	β 2.32<br>α 1.65	br. dd 12.6, 3.7<br>m	β 2.44<br>α 1.73	m<br>m	6β, 15α, 15β<br>15α	29.2	30.7	CH_2	
8	-		-			125.9	127.4	C	6α, 7β, 9, 15β
9	1.61	m	1.08	m	5, 12α	48.7	50.7	CH	7β, 11α, 12β, 19
10	-		-			36.9	38.7	C	6β, 9, 19
11	α 1.55<br>β 1.41	m<br>m	α 1.61<br>β 1.51	m<br>m	12α, 12β<br>1β, 12β, 18	19.5	21.1	CH_2	9
12	β 1.87<br>α 1.04	ddd 12.2, 3.2, 3.2<br>m	β 1.93<br>α 1.09	ddd 12.2, 3.4, 3.3<br>m	11α, 11β, 12α, 17, 21<br>9, 11α	36.9	38.3	CH_2	9, 18
13	-		-			42.2	43.8	C	11β, 15β, 16α, 17, 18
14	-		-			141.4	143.3	C	7α, 9, 12β, 15α, 15β, 16α, 16β, 18
15	α 2.18<br>β 2.11	br. dd 16.7, 10.2<br>m	2.19	m<br>m	7α, 7β, 16α, 16β<br>7β, 16α, 16β	25.3	26.6	CH_2	
16	α 1.71<br>β 1.28	dddd 13.1, 9.6, 7.3, 2.3<br>m	α 1.74<br>β 1.32	m<br>m	15α, 15β, 17<br>15α, 15β	26.6	28.1	CH_2	15β
17	1.02	m	1.03	m	12α, 16α, 21	56.3	58.2	CH	15α, 18, 20, 21
18	0.78	s	0.82	s	11β, 19, 20	18.1	18.7	CH_3	17
19	0.64	s	0.74	s	2β, 4, 18	13.7	14.4	CH_3	
20	1.37	m	1.39	m	18	34.1	35.9	CH	21, 23a
21	0.86	d 6.6	0.88	d 6.7	12β, 17	19.0	19.6	CH_3	23b
22	1.33<br>0.98	m<br>m	a 1.41<br>b 1.02	m<br>m	24	32.5	34.1	CH_2	21

Table S1. *Cont.*

23	1.48<br>1.15	dddd 12.4, 12.0, 6.7, 4.3<br>dddd 12.0, 10.6, 6.6, 4.8	a 1.51<br>b 1.19	m<br>m	24, 26b<br>24	31.3	33.4	CH_2	24, 28
24	2.50	m	2.62	ddq 7.0, 7.0, 7.0	22a, 23a, 23b	35.0	36.6	CH	23b, 26a, 26b, 28
25	-		-			151.4	152.7	C	23a, 23b, 24, 26a, 26b, 28
26	5.53<br>5.17	s<br>s	a 5.68<br>b 5.26	s<br>br. s	NH<br>23a, 28	113.9	115.8	CH_2	24
27	-		-			166.7	170.3	C	24, 26a, 26b, 30, NH
28	0.99	d 6.9	1.08	d 6.9	26b	19.7	19.8	CH_3	23a, 23b, 24
29	0.87	d 6.3	1.02	d 6.3	3	15.5	16.0	CH_3	
30	4.60	d 5.8	5.25	s	37	58.2	59.9	CH	33, 37, NH
31	-		-			170.4	173.3	C	30, NH
32	-		-			134.9	132.9	C	
33	7.18	d 8.6	7.36	d 8.7		127.5	129.4	CH	30, 37
34	6.74	d 8.6	6.82	d 8.7	38	112.7	114.6	CH	33, 36
35	-		-			157.5	160.3	C	33, 34, 36, 37, 38
36	6.74	d 8.6	6.82	d 8.7	38	112.7	114.6	CH	34, 37
37	7.18	d 8.6	7.36	d 8.7	30, NH	127.5	129.4	CH	30, 33
38	3.67	s	3.75	s	34, 36	55.0	55.7	CH_3	
NH	7.78	d 5.8			26a, 37		-		

Table S2. ^{1}H (600 MHz) and ^{13}C (150 MHz) NMR chemical shifts (DMSO-d_6 and CD_3OD), NOESY and HMBC correlations of compound **2**.

No.	DMSO-d_6		CD_3OD		NOESY	DMSO-d_6	CD_3OD	Type	HMBC
	^{1}H (δ)	m (*J*)	^{1}H (δ)	m (*J*)		^{13}C (δ)	^{13}C (δ)		($^{13}C\rightarrow{}^{1}H$)
1	β 1.56<br>α 1.02	m<br>m	β 1.71<br>α 1.17	ddd 13.3, 3.5, 3.5<br>m	2α, 2β, 19<br>2α, 3, 5	35.6	37.2	CH_2	2α, 19
2	α 2.09<br>β 1.28	dm 12.2<br>m	α 2.27<br>β 1.56	m<br>m	1α, 1β, 3<br>1β, 19	28.1	29.3	CH_2	1β
3	3.50	ddd 11.2, 10.0, 4.8	3.85	ddd 11.2, 10.0, 4.8	1α, 2α, 5, 29	80.2	85.1	CH	1β, 29
4	1.20	m	1.42	m	6β, 19, 29	36.8	38.4	CH	6β, 29, 5
5	1.22	m	1.38	ddd 11.5, 9.0, 2.5	1α, 3, 6α, 9, 29, 39	44.1	45.7	CH	1α, 4, 7, 19, 29
6	α 1.83<br>β 1.07	m<br>m	α 1.98<br>β 1.20	ddd 14.6, 2.9, 2.5<br>m	5, 39<br>4, 7, 19	30.4	31.4	CH_2	5, 7
7	3.94	br. s	4.08	dd 2.9, 2.8	6β, 15β	73.3	75.6	CH	6α, 9, 39
8	-		-			124.5	125.8	C	6α, 9, 11β, 15α
9	1.87	m	1.95	m	5, 11α, 39	43.4	45.3	CH	1β, 7, 12β, 19
10	-		-			37.1	38.6	C	5, 6α, 9, 19
11	α 1.56<br>β 1.36	m<br>m	α 1.63<br>β 1.48	dddd 13.9, 7.5, 3.4, 3.4<br>m	9, 12α, 12β<br>12β, 18, 19	19.8	20.5	CH_2	9, 12β

Table S2. *Cont.*

12	β 1.89<br>α 1.04	m<br>m	β 1.96<br>α 1.12	m<br>m	11α, 11β, 18, 21<br>11α, 39	36.6	38.2	CH_2	9, 18
13	-		-			42.8	44.6	C	11β, 12α, 15α, 17, 18
14	-		-			147.9	150.6	C	7, 9, 12β, 15α, 15β, 16α, 16β
15	α 2.37<br>β 2.21	ddd 17.6, 9.5, 8.7<br>br. dd 17.6, 12.4	α 2.43<br>β 2.25	ddd 17.6, 9.5, 8.7<br>m	16α, 17, 39<br>7, 16β, 18	25.0	26.4	CH_2	
16	α 1.76<br>β 1.31	m<br>m	α 1.80<br>β 1.34	dddd 13.0, 9.5, 7.1, 2.3<br>m	15α, 17, 22a, 22b<br>15β, 18	26.4	27.9	CH_2	15α, 17
17	1.05	m	1.10	m	15α, 16α, 21	56.6	58.4	CH	16β, 18, 21, 20, 22a, 22b
18	0.80	s	0.85	s	11β, 12β, 15β, 16β, 20	17.4	18.1	CH_3	12α
19	0.63	s	0.74	s	1β, 2β, 4, 6β, 11β	12.9	13.6	CH_3	1α
20	1.38	m	1.41	m	18, 21	34.1	35.8	CH	21, 22b, 23a
21	0.89	d 6.4	0.91	d 6.6	12β, 17, 20, 23b	19.0	19.6	CH_3	
22	a1.36<br>b0.97	m<br>m	a1.41<br>b1.02	m<br>m	16α, 24, 28<br>16α, 23a	32.5	33.9	CH_2	20, 21, 23b, 24
23	a1.49<br>b1.17	m<br>m	a1.53<br>b1.22	m<br>m	22b, 24, 26b, 28<br>21, 24, 26b	31.5	33.3	CH_2	24, 28, 20, 22a
24	2.50	m	2.62	ddq 6.5, 6.5, 6.5	22a, 23a, 23b, 26b, 28	35.0	36.5	CH	23a, 23b, 26a, 26b, 28
25	-		-			151.3	152.6	C	23a, 23b, 24, 26a, 26b, 28
26	a5.53<br>b5.17	s<br>br. s	a5.69<br>b5.27	s<br>br. s	 23a, 23b, 24, 28	114.0	116.0	CH_2	24
27	-		-			166.7	170.9	C	26a, 26b, NH
28	1.00	d 6.9	1.08	d 6.9	22a, 23a, 24, 26b	19.0	20.0	CH_3	24
29	0.84	d 5.8	1.01	d 6.0	3, 4, 5	15.4	15.8	CH_3	5
30	4.57	d 5.0	5.24	s	33, 37	58.2	59.9	CH	33, 37, NH
31	-		-			170.5	176.8	C	30, NH
32	-		-			134.9	134.0	C	30, 34, 36
33	7.18	d 8.4	7.36	d 8.7	30, 34	127.5	129.4	CH	30, 37
34	6.74	d 8.4	6.83	d 8.7	33, 38	112.7	114.6	CH	36
35	-		-			157.5	160.3	C	33, 34, 36, 37, 38
36	6.74	d 8.4	6.83	d 8.7	37, 38	112.7	114.6	CH	34
37	7.18	d 8.4	7.36	d 8.7	30, 36	127.5	129.4	CH	30, 33
38	3.69	s	3.75	s	34, 36	55.0	55.7	CH_3	
39	3.03	s	3.16	s	5, 6α, 9, 12α, 15α	53.5	54.6	CH_3	
NH	7.78	d 5.0					-		

Table S3. Conformational analysis of (24*S*,30*S*)-**1** at the B3LYP/6-31G** level in the gas phase.

# [a]	ΔE [b]	P% [c]
1	10.20	1.6
2	11.63	0.9
3	34.93	0.0
4	26.12	0.0
5	32.90	0.0
6	26.12	0.0
7	0.00	97.5
8	26.12	0.0
9	27.92	0.0

[a]: conformer number; [b]: relative energy, zero point vibrational energy was included; [c]: conformational distribution.

Table S4. Important transition states, related rotatory strengths, and oscillator strengths of (24*S*,30*S*)-**1** at the B3LYP/6-31G* level in the gas phase.

Transitions	*ΔE* [a] (eV)	λ [b] (nm)	*f* [c]	R_{len} [d]
183→186, 180→186	4.82	257.0	0.005	−12.58
182→187, 183→188	5.37	230.9	0.049	38.82
183→188, 182→187	5.39	230.2	0.110	60.84
182→188	5.57	222.5	0.027	9.35
178→186, 176→186, 180→187, 179→186	5.72	216.9	0.022	5.06
180→187, 178→186, 176→186	5.75	215.6	0.039	−24.97
181→188, 183→189, 180→188, 180→187	5.82	212.9	0.004	−14.31
183 →189, 180→188	5.88	210.8	0.010	−7.00
178→188, 179→188, 179→186, 176→188	6.04	205.2	0.006	−12.20
182→189, 176→188	6.15	201.6	0.198	83.36
182→189	6.16	201.3	0.172	−136.69
184→190	6.17	200.9	0.005	−5.32

[a] Excited energy; [b] Wavelength; [c] Oscillator strength; [d] Rotatory strength in length form (10^{-40}cgs).

Table S5. Experimental and calculated NMR chemical shifts of **1**.

No.[a]	Atom [b]	δ_c [c]	24*S*,30*S*		24*R*,30*S*		δ_i [f]
			δ_c [d]	Δ [e]	δ_c [d]	Δ [e]	
1	C	35.8	40.1	4.3	40.5	4.7	0.4
2	C	28.2	31.9	3.7	32.5	4.3	0.6
3	C	80.1	75.6	4.5	76.0	4.1	0.4
4	C	37.2	40.7	3.5	41.0	3.8	0.3
5	C	50.7	51.4	0.7	51.6	0.9	0.2
6	C	24.7	24.8	0.1	25.0	0.3	0.1
7	C	29.2	31.8	2.6	31.8	2.6	0.0
8	C	125.9	131.2	5.3	132.6	6.7	1.4
9	C	48.7	55.9	7.2	56.6	7.9	0.6
10	C	36.9	39.7	2.8	39.5	2.6	0.2
11	C	19.5	23.3	3.8	24.4	4.9	1.1

Table S5. *Cont.*

12	C	36.9	40.0	3.1	41.4	4.5	1.5
13	C	42.2	47.5	5.3	47.7	5.5	0.2
14	C	141.4	136.7	4.7	136.9	4.5	0.2
15	C	25.3	27.4	2.1	27.5	2.2	0.2
16	C	26.6	32.4	**5.8**	31.6	5.0	0.8
17	C	56.3	54.2	2.1	52.0	4.3	2.2
18	C	18.1	20.5	2.4	20.3	2.2	0.1
19	C	13.7	17.0	3.3	17.5	3.8	0.5
20	C	34.1	39.7	**5.6**	39.4	5.3	0.2
21	C	19.0	16.9	2.1	19.3	0.3	2.4
22	C	32.5	36.5	4.0	35.0	2.5	1.5
23	C	31.3	33.0	1.7	34.9	3.6	1.9
24	C	35.0	43.1	8.1	48.4	13.4	5.3
25	C	151.4	155.0	3.6	152.6	1.2	2.3
26	C	113.9	106.2	**7.7**	107.4	**6.5**	1.2
27	C	166.7	161.2	5.5	159.9	6.8	1.3
**28**	**C**	**19.7**	**22.7**	**3.0**	**18.0**	**1.7**	**4.7**
29	C	15.5	15.6	0.1	16.0	0.5	0.4
30	C	58.2	59.6	1.4	61.4	3.2	1.8
31	C	170.4	163.2	**7.2**	164.8	**5.6**	1.5
32	C	134.9	128.6	**6.3**	130.9	**4.0**	2.2
33	C	127.5	125.8	1.7	126.5	1.0	0.8
34	C	112.7	103.6	**9.1**	103.8	**8.9**	0.2
35	C	157.5	150.8	**6.7**	151.3	**6.2**	0.5
36	C	112.7	110.8	1.9	111.7	1.0	0.9
37	C	127.5	119.5	**8.0**	120.3	**7.2**	0.8
38	C	55.0	52.0	3.0	52.0	3.0	0.1

[a] Atom numbering; [b] atom name; [c] Experimentally and [d] Theoretically observed chemical shifts; [e] Difference between experimentally and theoretically observed chemical shifts; [f] Difference between calculated chemical shifts of two configurations. Calculation was performed at the B3LYP/6-31G** level in the gas phase.

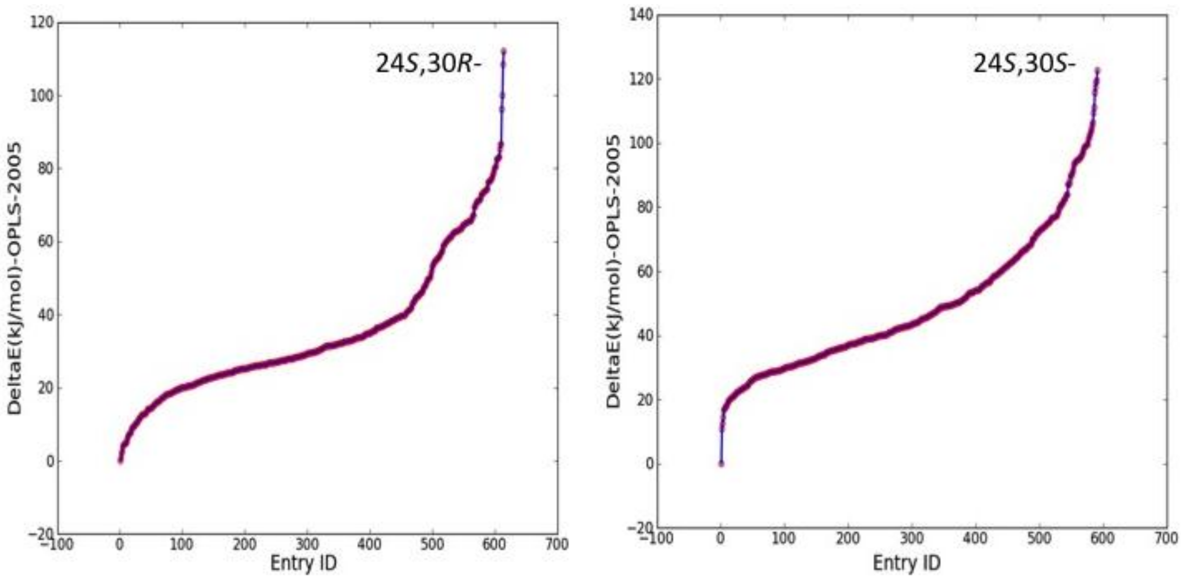

Figure S1. Random conformational search of (24*S*,30*R*) and (24*S*,30*S*)**-1** with an energy window of 130 kJ/mol.

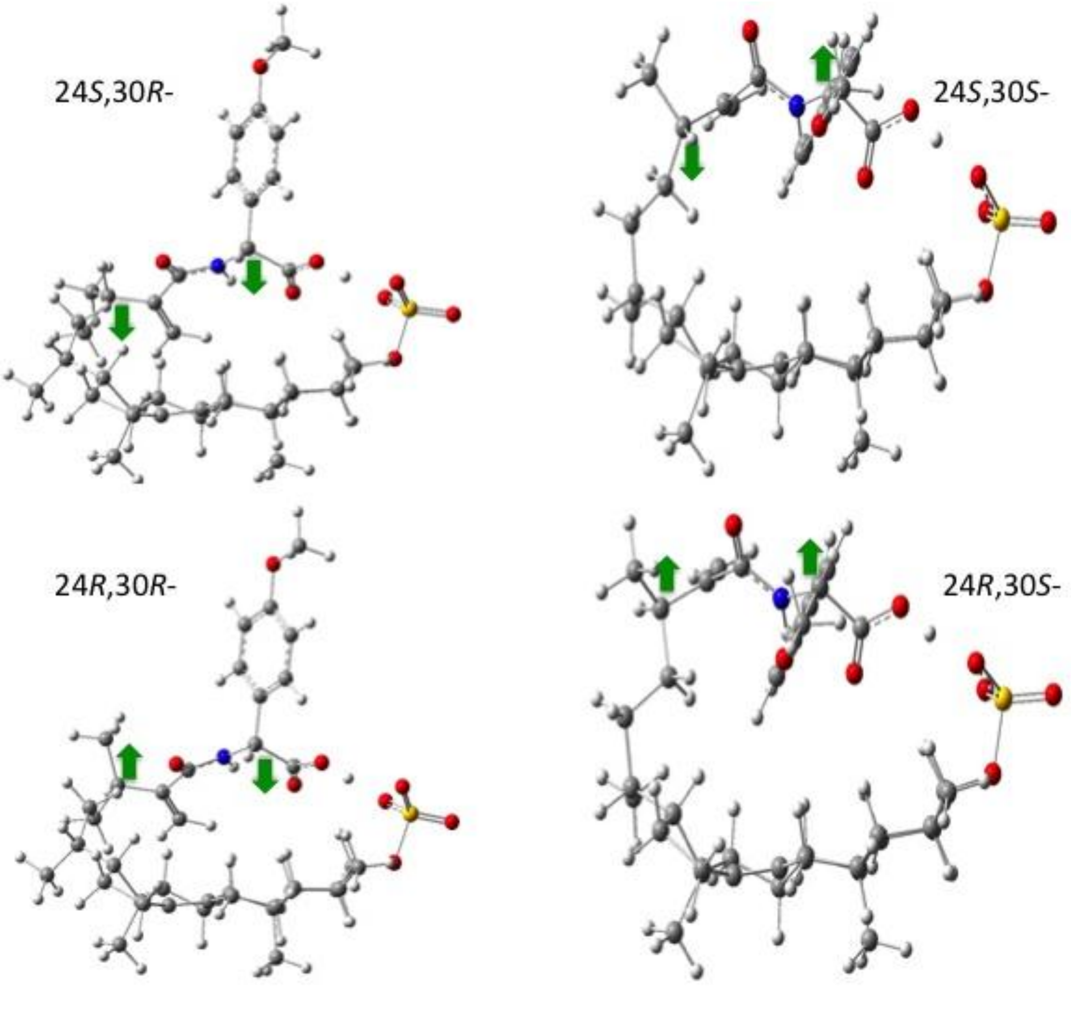

Figure S2. Optimized geometries of predominant conformers of **1** at the B3LYP/6-31G** level in the gas phase.

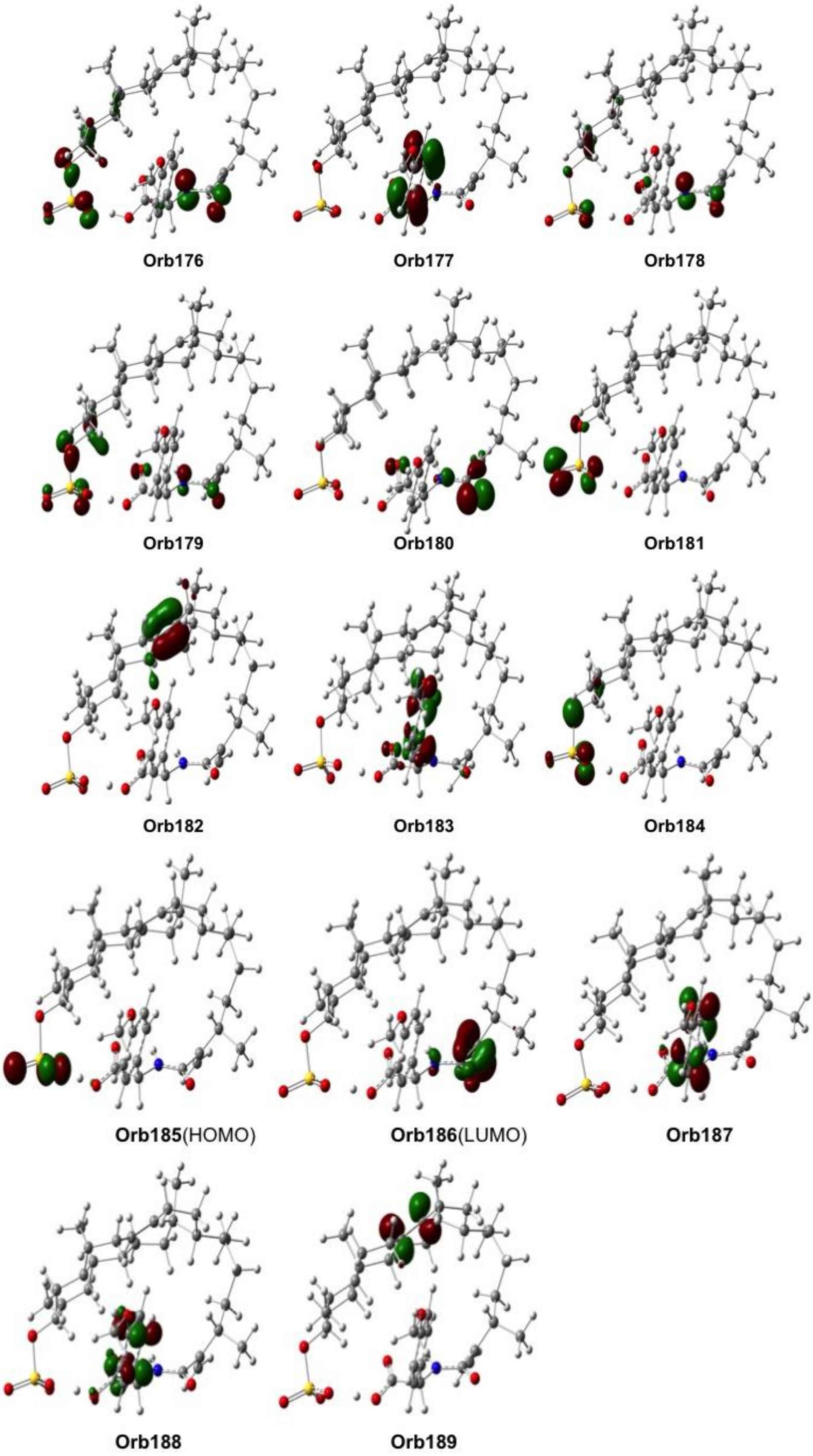

Figure S3. Molecular orbitals involved in the key transitions in the calculated ECD spectrum of (24*S*,30*S*)-**1** at the B3LYP/6-31G** level in the gas phase.

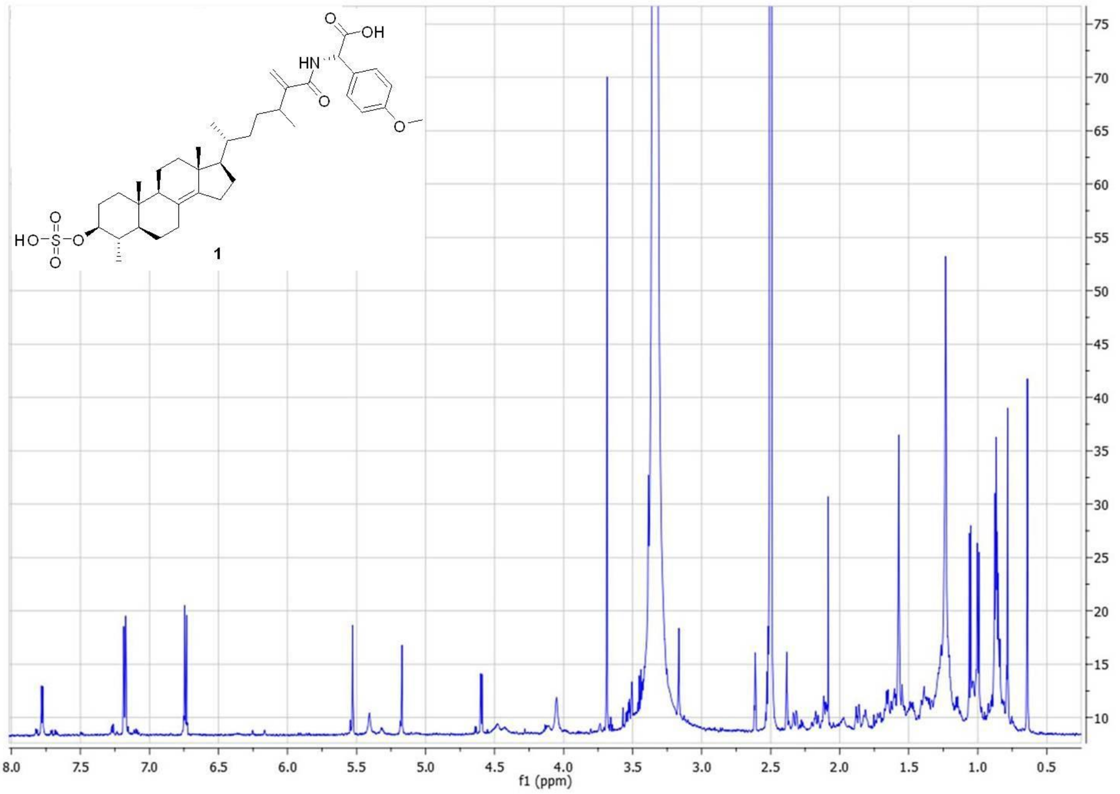

Figure S4. ^{1}H-NMR spectrum (600 MHz, DMSO-d_6) of compound **1**.

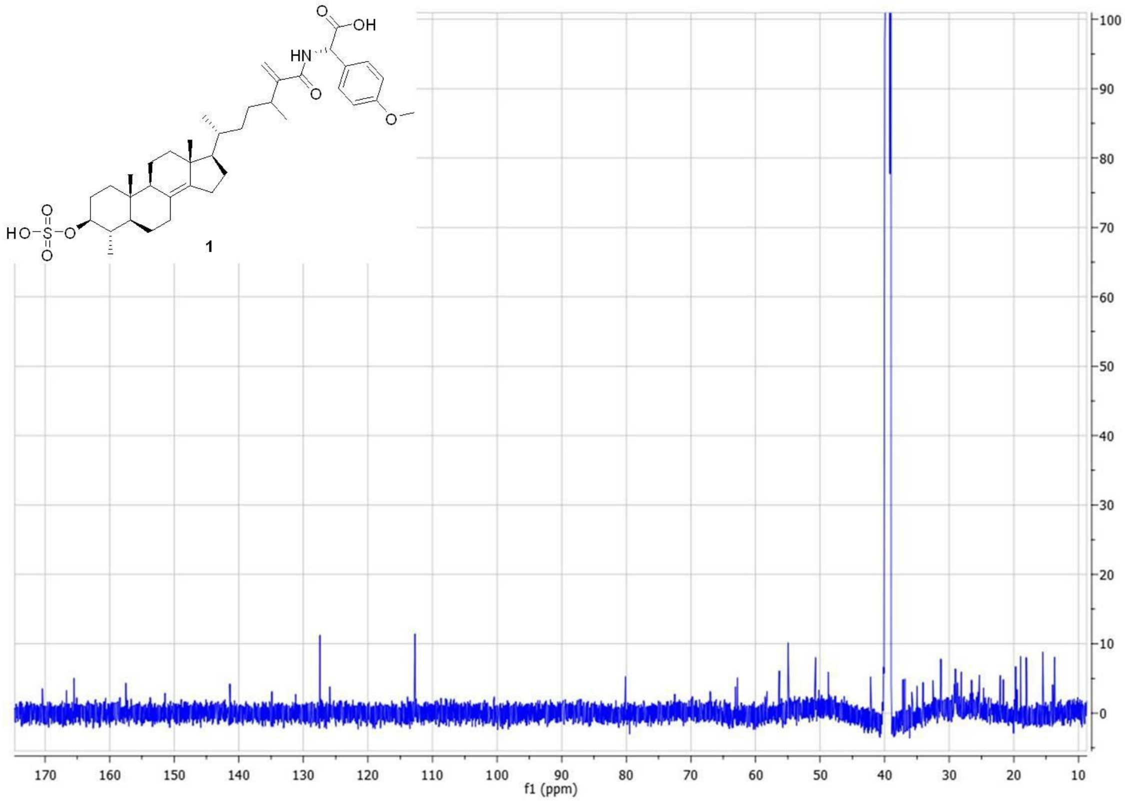

Figure S5. ^{13}C-NMR spectrum (150 MHz, DMSO-d_6) of compound **1**.

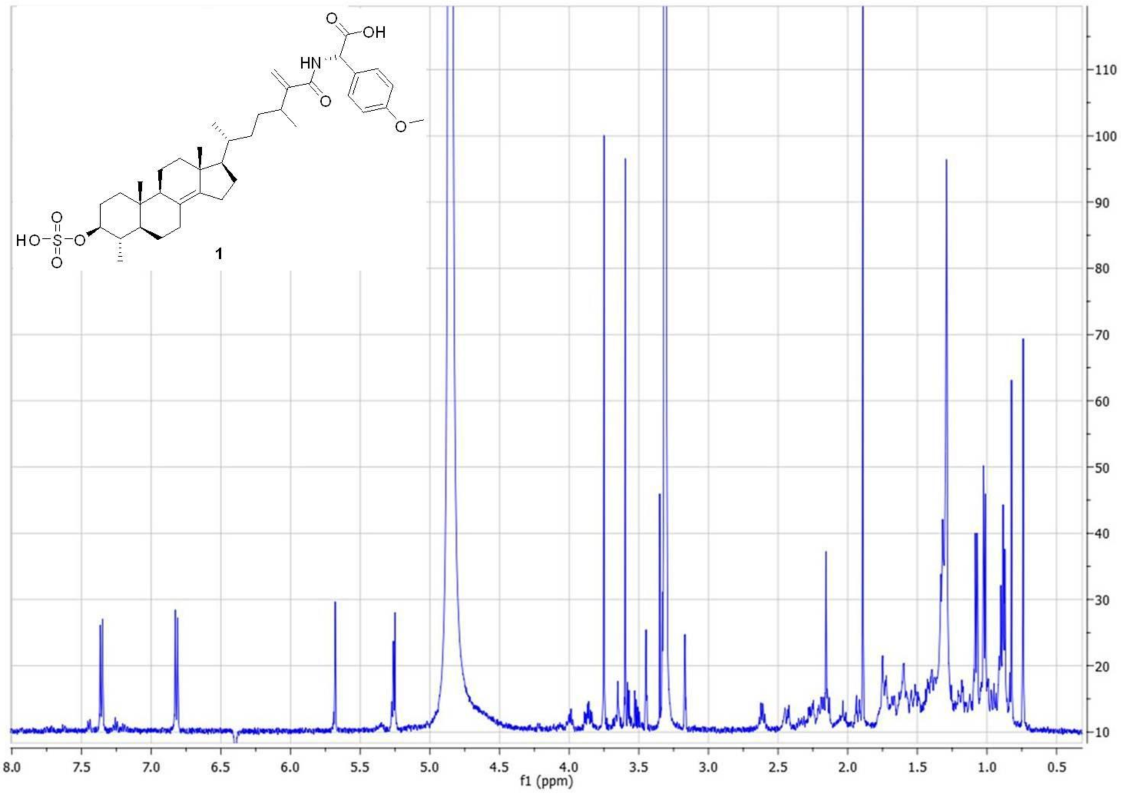

Figure S6. ^{1}H-NMR spectrum (600 MHz, CD_3OD) of compound **1**.

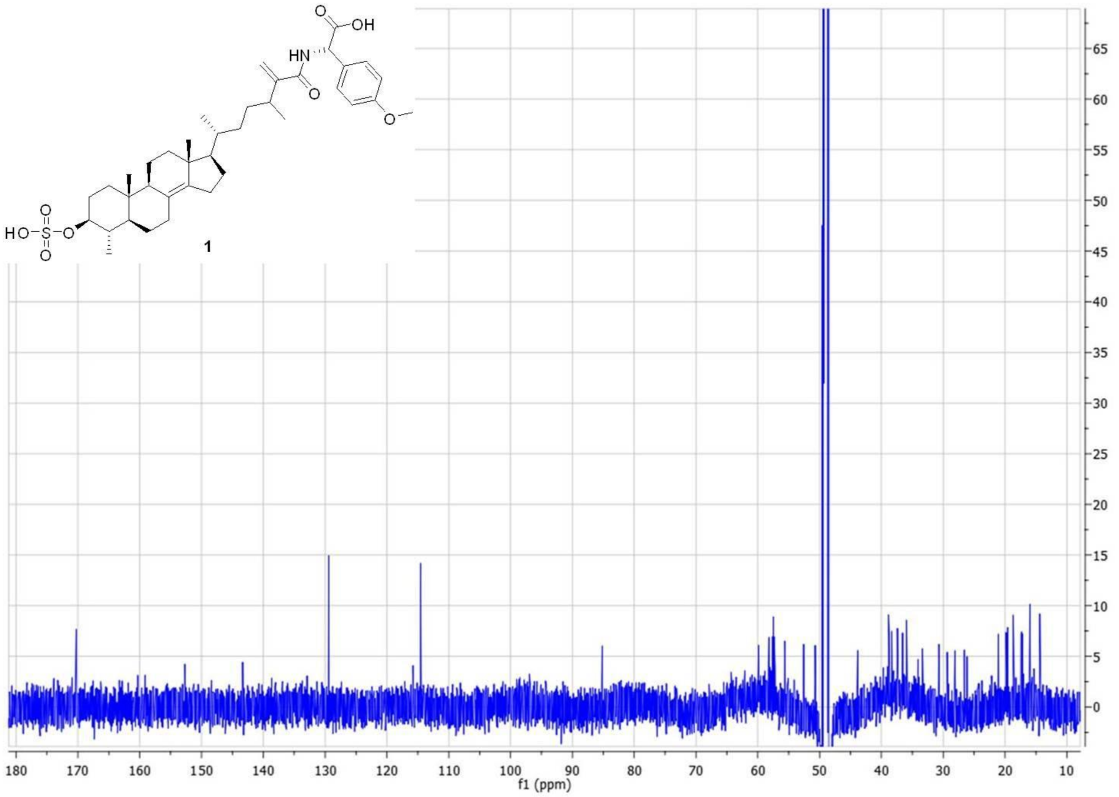

Figure S7. ^{13}C NMR spectrum (150 MHz, CD_3OD) of compound **1**.

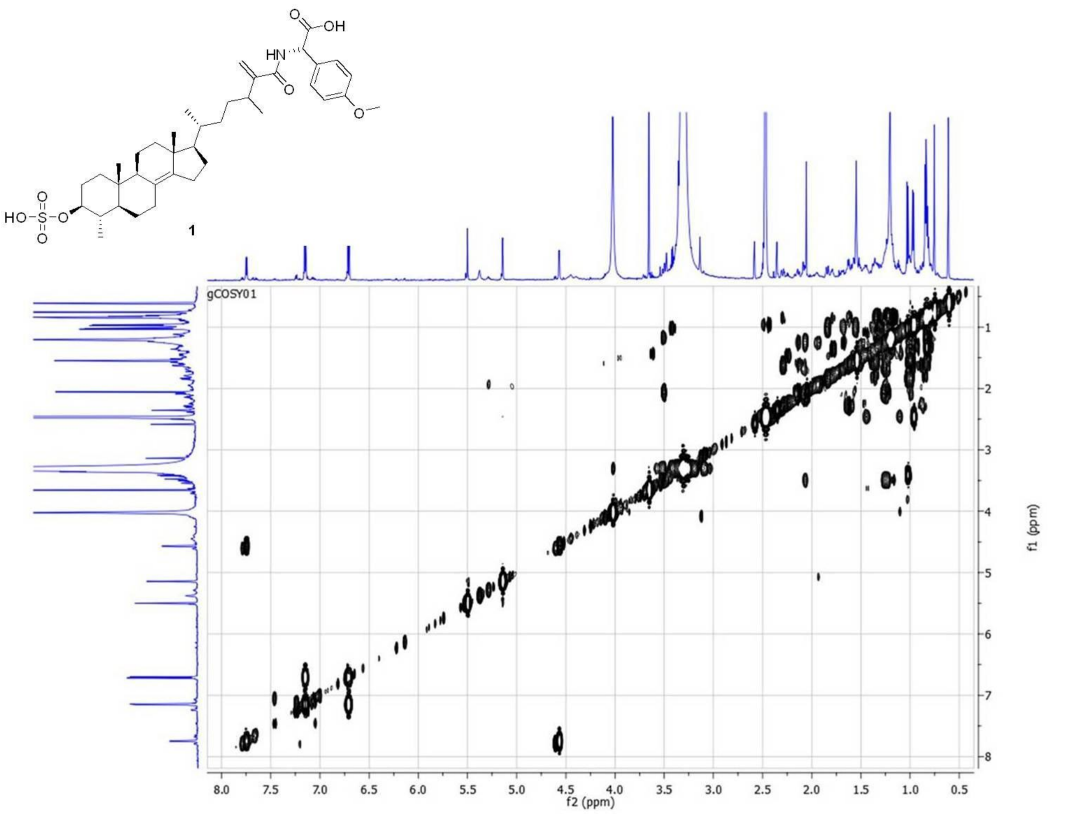

Figure S8. ^{1}H-^{1}H COSY spectrum (DMSO-*d*6) of compound **1**.

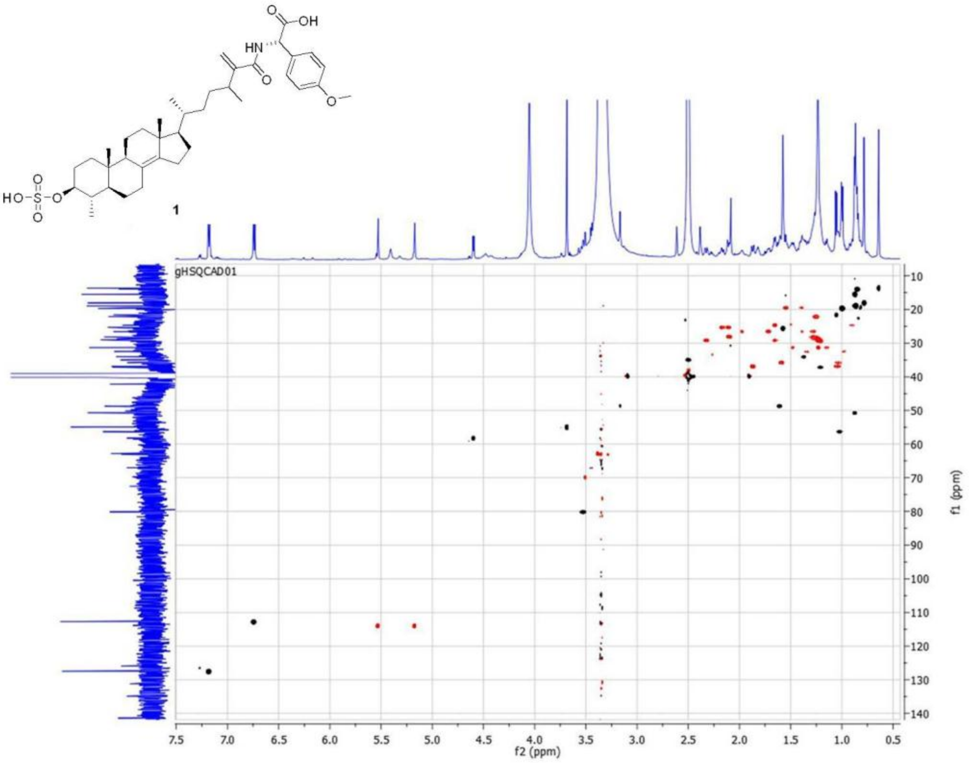

Figure S9. HSQC spectrum (DMSO-*d*6) of compound **1**.

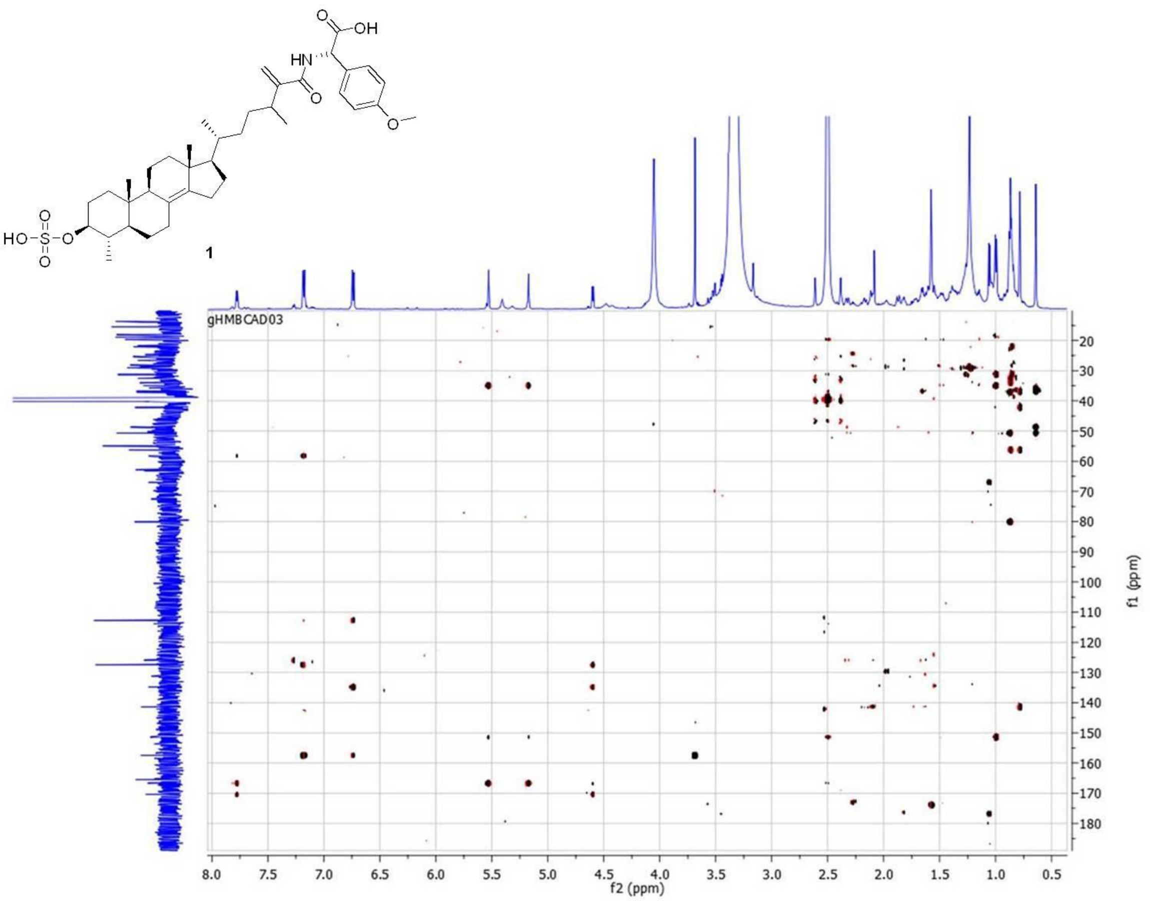

Figure S10. HMBC spectrum (DMSO-d_6) of compound **1**.

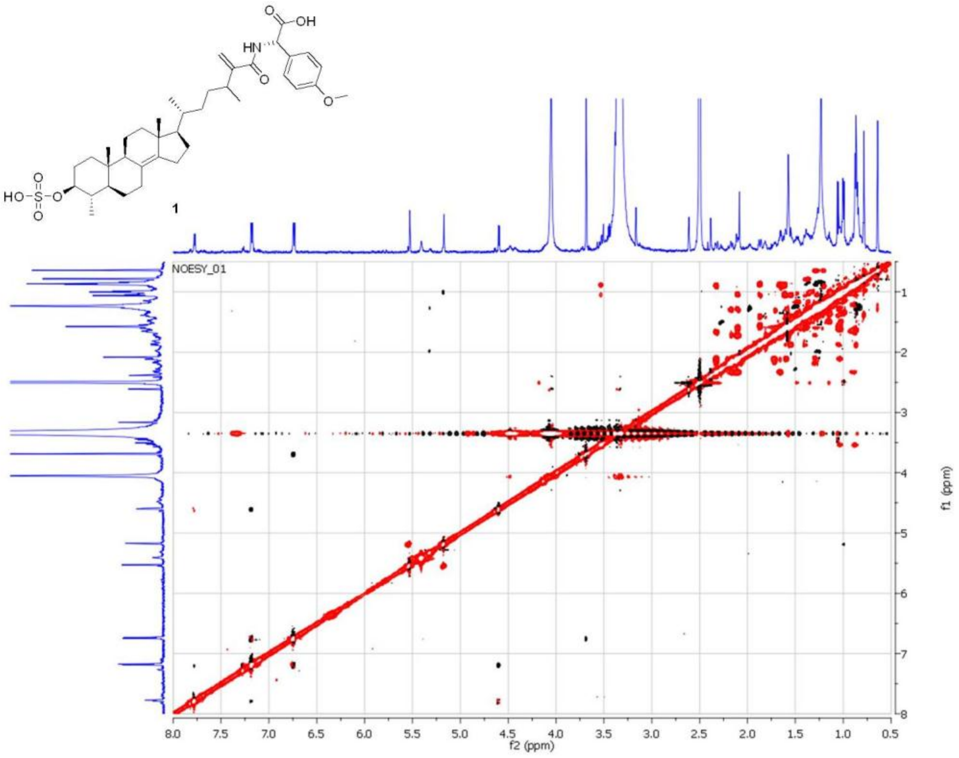

Figure S11. NOESY spectrum (DMSO-d_6) of compound **1**.

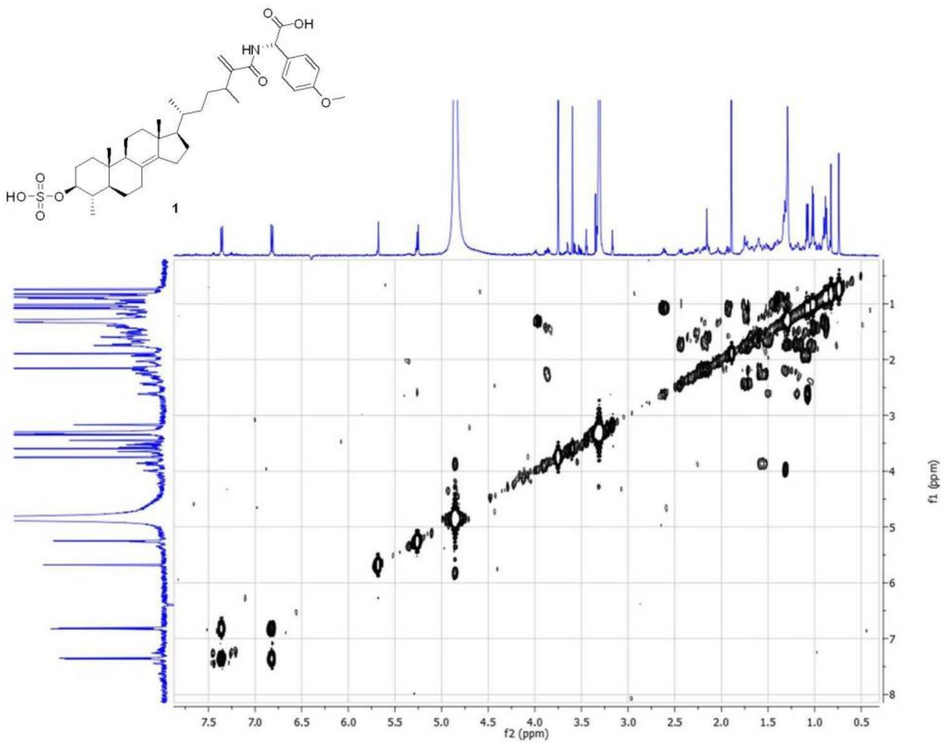

Figure S12. ^{1}H-^{1}H COSY spectrum (CD_3OD) of compound **1**.

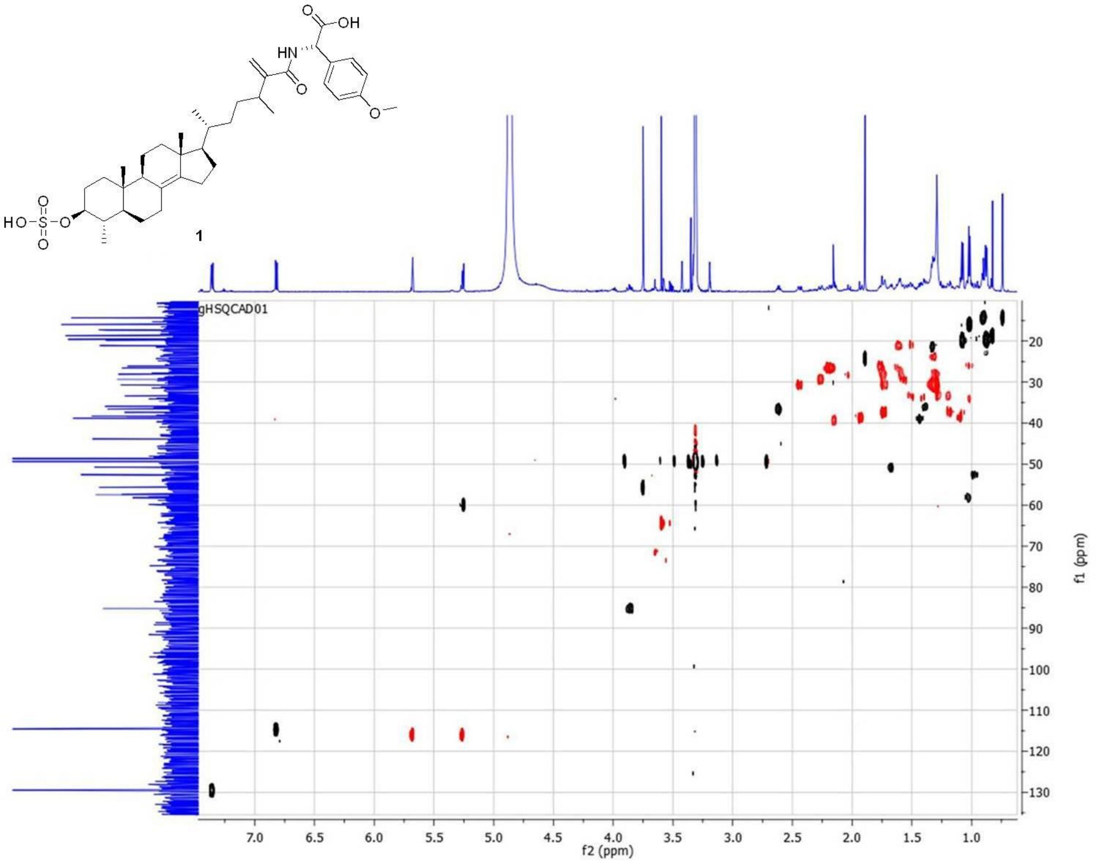

Figure S13. HSQC spectrum (CD_3OD) of compound **1**.

Qualitative Compound Report

**Data File**	Poly1-neg_2014-07-01.d	**Sample Name**	Poly1
**Sample Type**	Sample	**Position**	P1-A4
**Instrument Name**	Instrument 1	**User Name**	
**Acq Method**	VsmyNegPoly.m	**Acquired Time**	01/07/2014 16:06:3
**IRM Calibration Status**	Success	**DA Method**	Default.m
**Comment**			

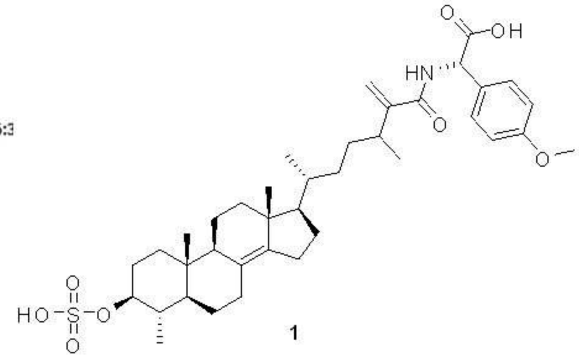

Sample Group **Info.**
Acquisition SW 6200 series TOF/6500 series
Version Q-TOF B.05.01 (B5125)

Compound Table

Compound Label	RT	Mass	Abund	Formula	Tgt Mass	Diff (ppm)	MFG Formula	DB Formula
Cpd 1: C38 H55 N O8 S	10.825	685.3646	546887	C38 H55 N O8 S	685.3648	-0.3	C38 H55 N O8 S	C38 H55 N O8 S

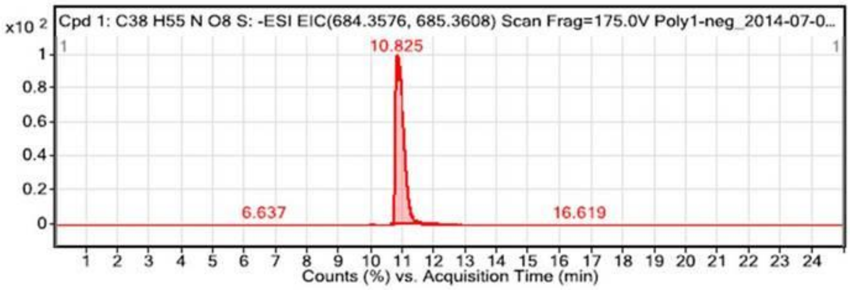

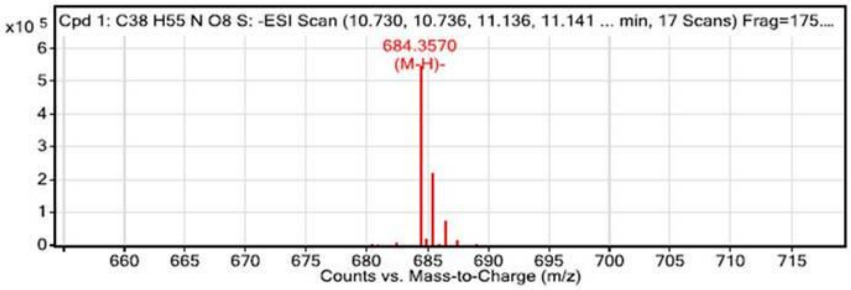

MS Spectrum Peak List

m/z	*Calc m/z*	Diff(ppm)	z	Abund	Ion
684.357	684.3576	0.75	-1	546886.5	(M-H)-
685.3611	685.3608	-0.34	-1	221727.02	(M-H)-
686.3613	686.3607	-0.81	-1	79107.63	(M-H)-
687.3623	687.3617	-0.84	-1	19181.14	(M-H)-
688.362	688.3632	1.67	-1	4155.32	(M-H)-
689.3617	689.365	4.72	-1	1045.77	(M-H)-

Figure S14. ESIMS spectrum of compound **1**.

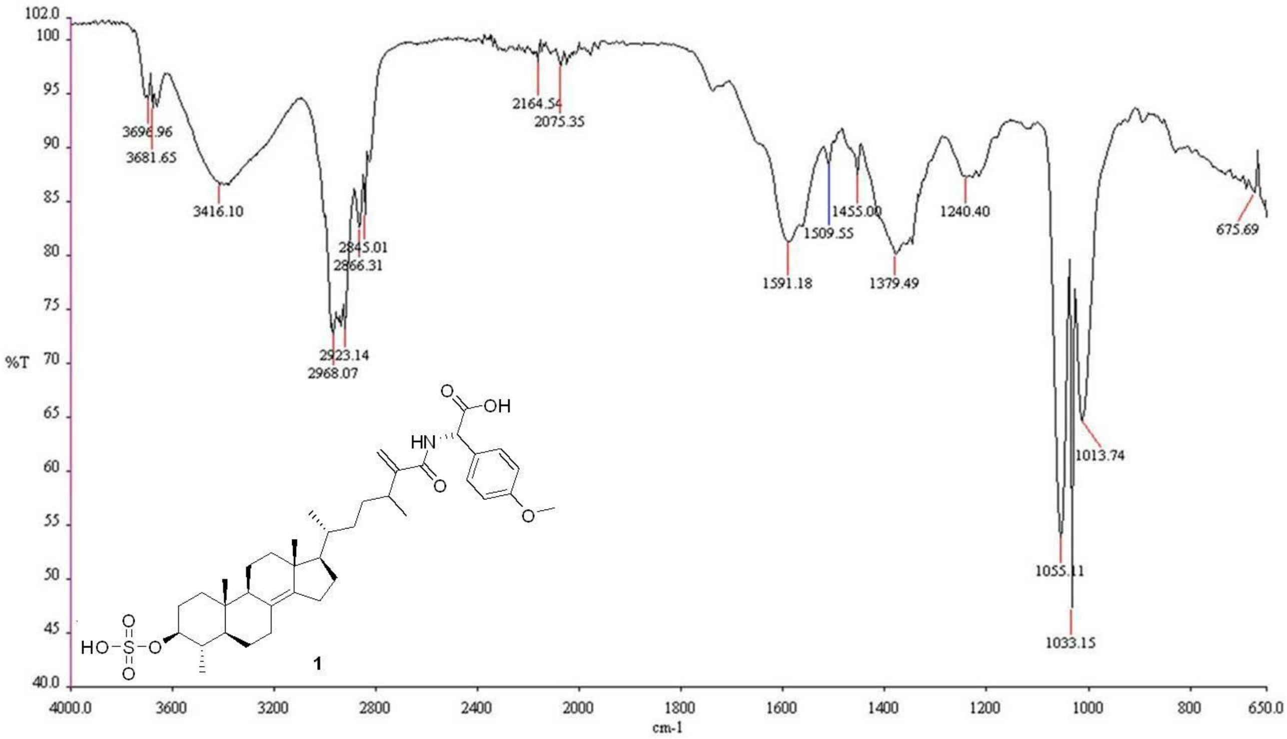

Figure S15. IR spectrum of compound **1**.

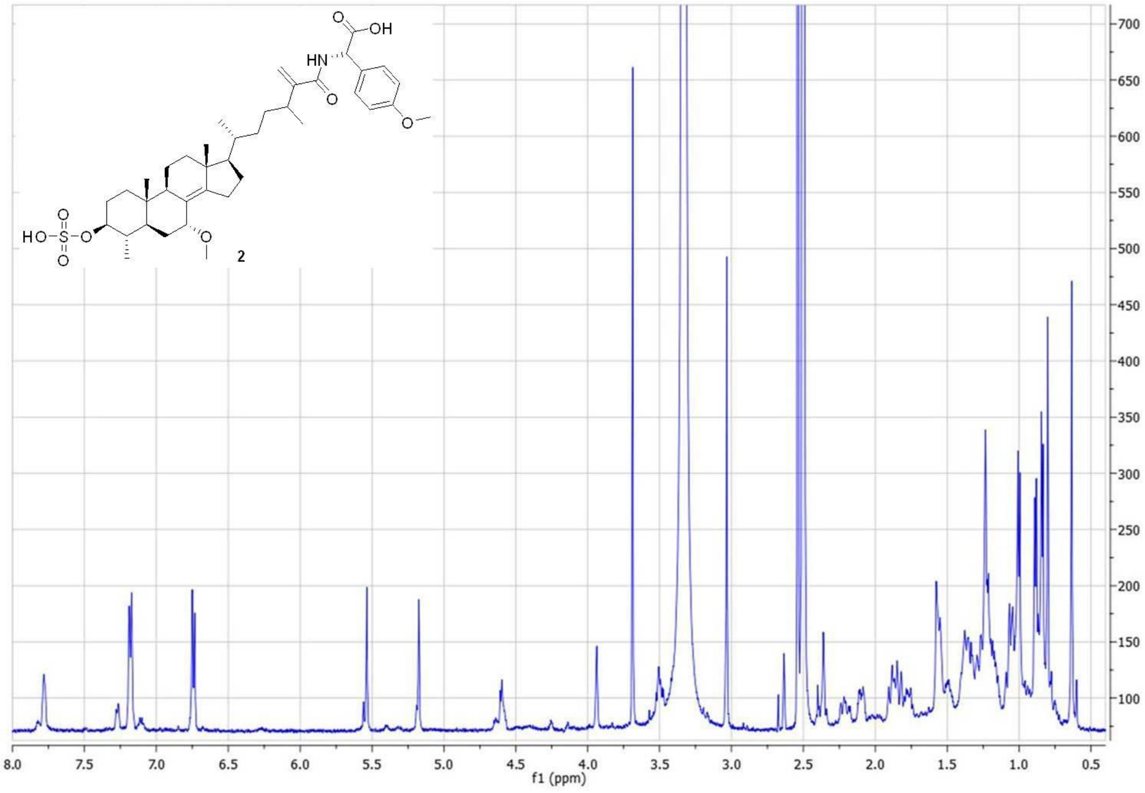

Figure S16. ^{1}H-NMR spectrum (600 MHz, DMSO-d_6) of compound **2**.

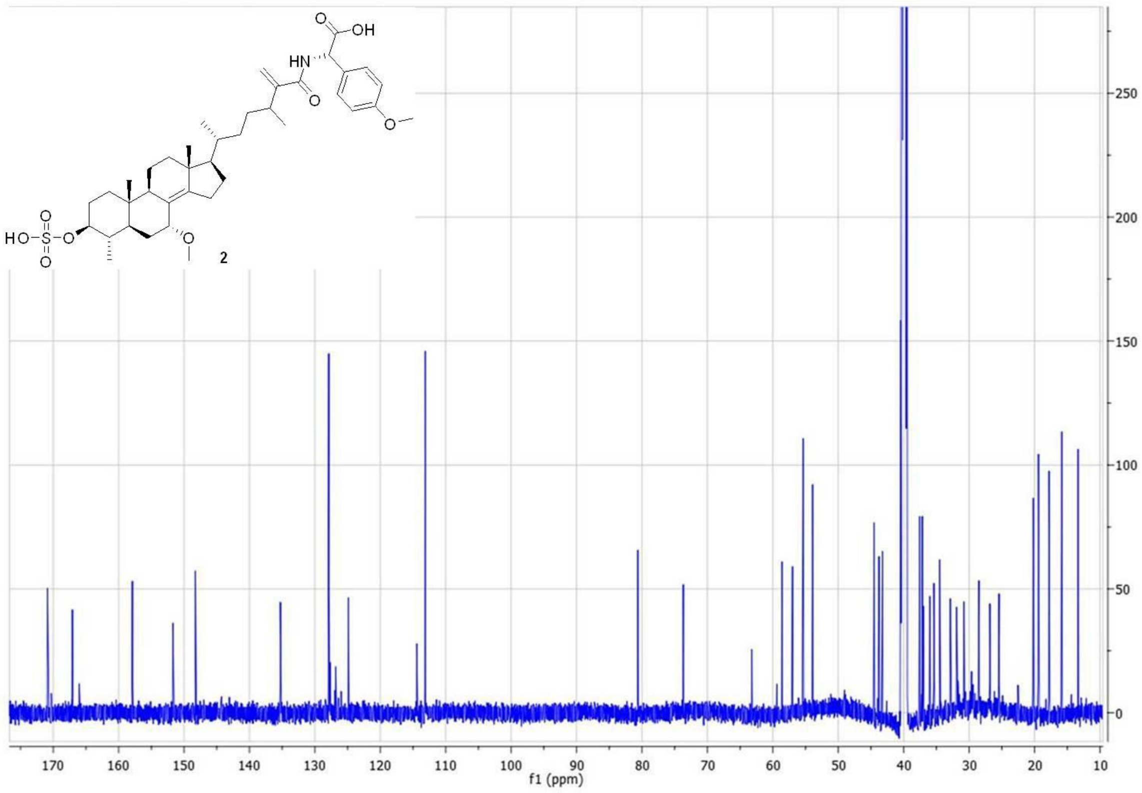

Figure S17. ^{13}C-NMR spectrum (150 MHz, DMSO-d_6) of compound **2**.

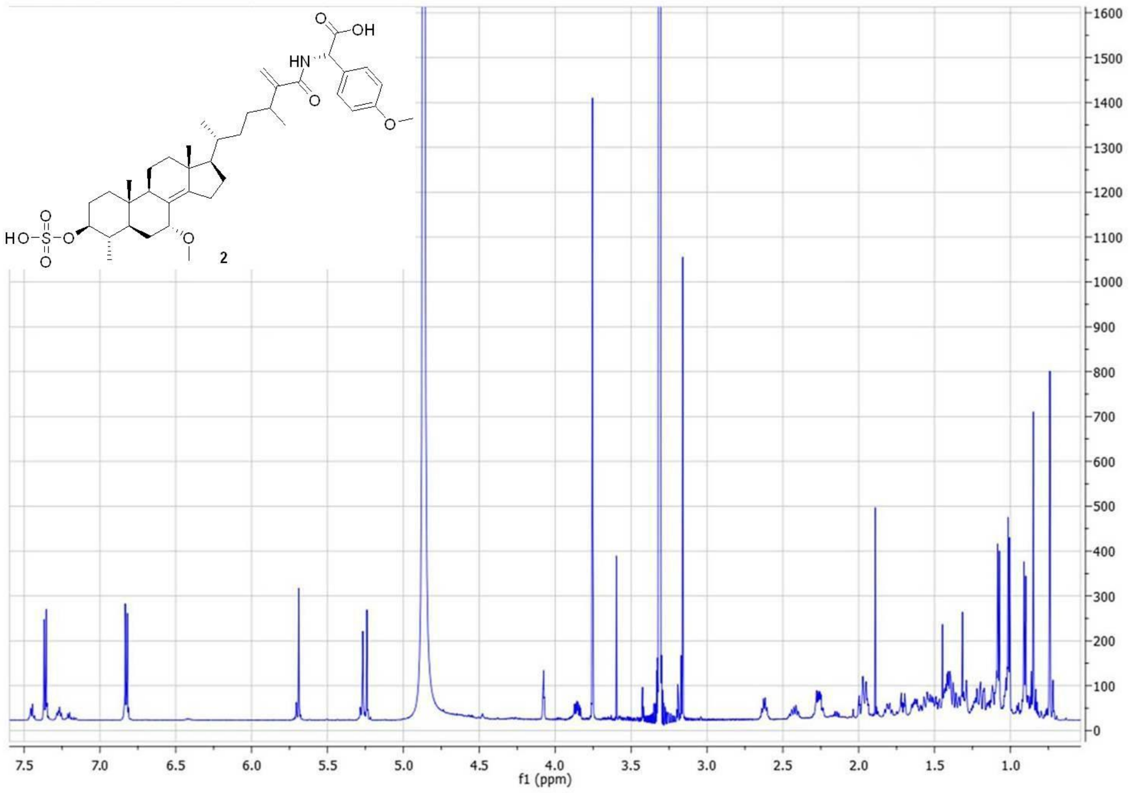

Figure S18. ^{1}H-NMR spectrum (600 MHz, CD_3OD) of compound **2**.

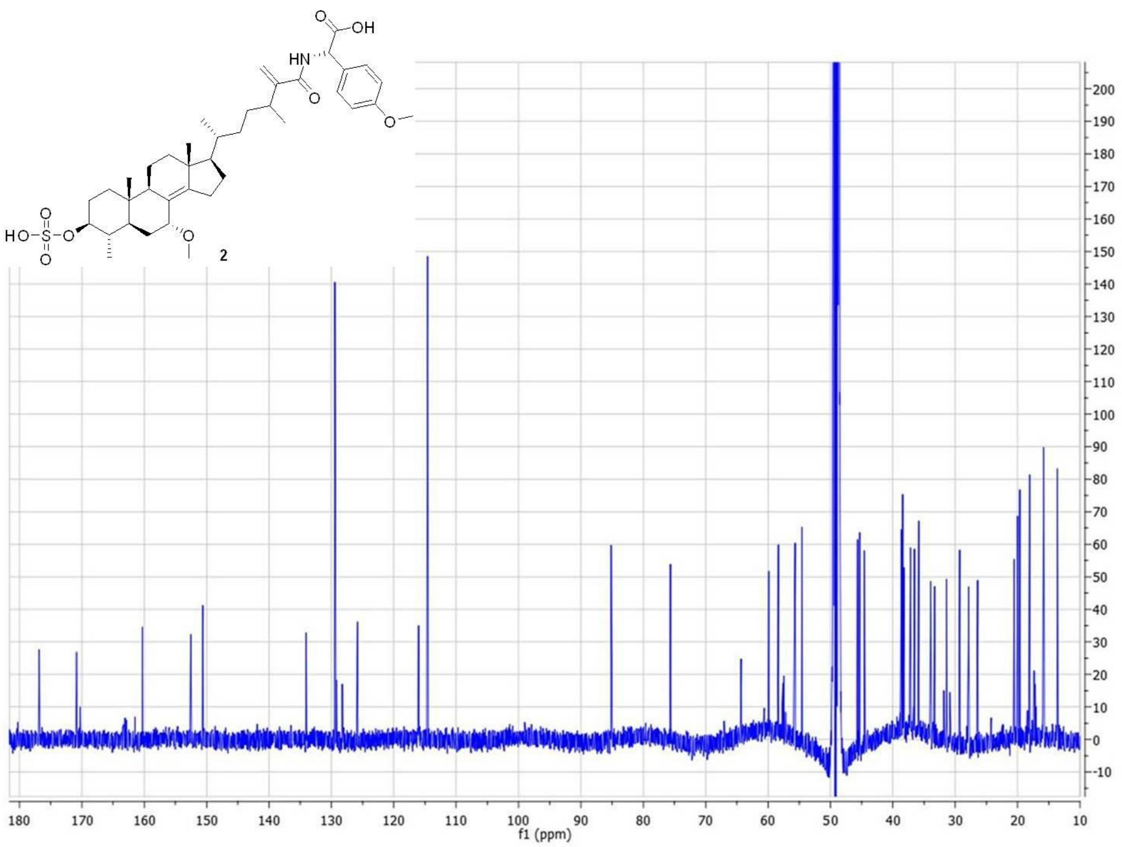

Figure S19. ^{13}C-NMR spectrum (150 MHz, CD_3OD) of compound **2**.

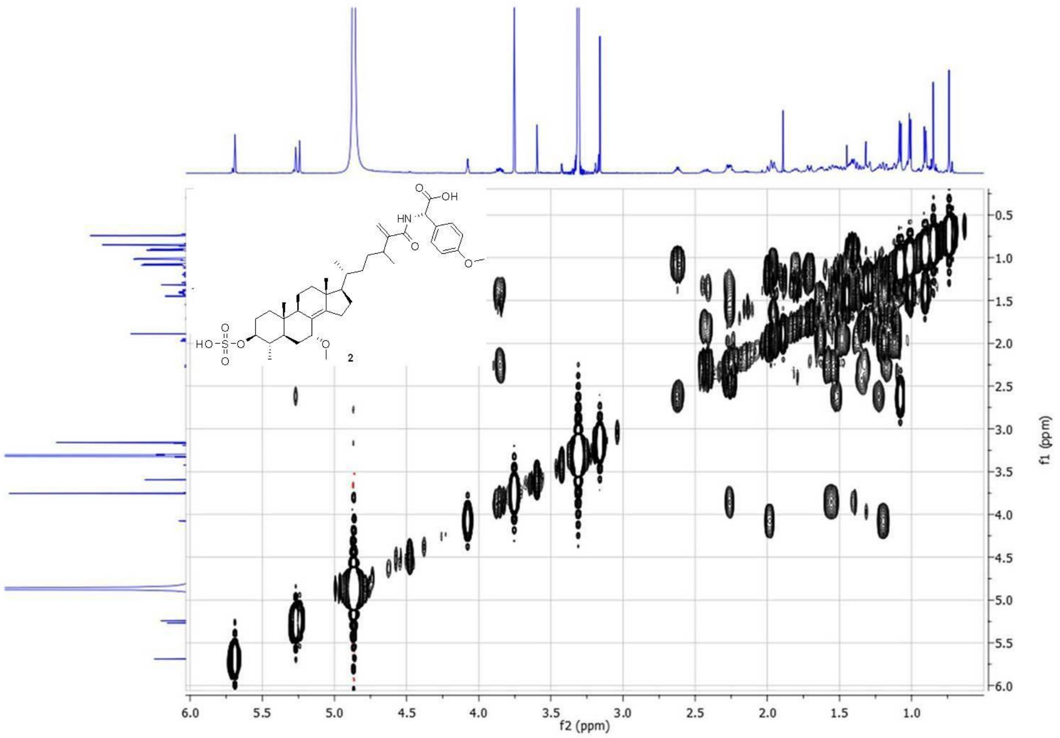

Figure S20. ^{1}H-^{1}H COSY spectrum (CD_3OD) of compound **2**.

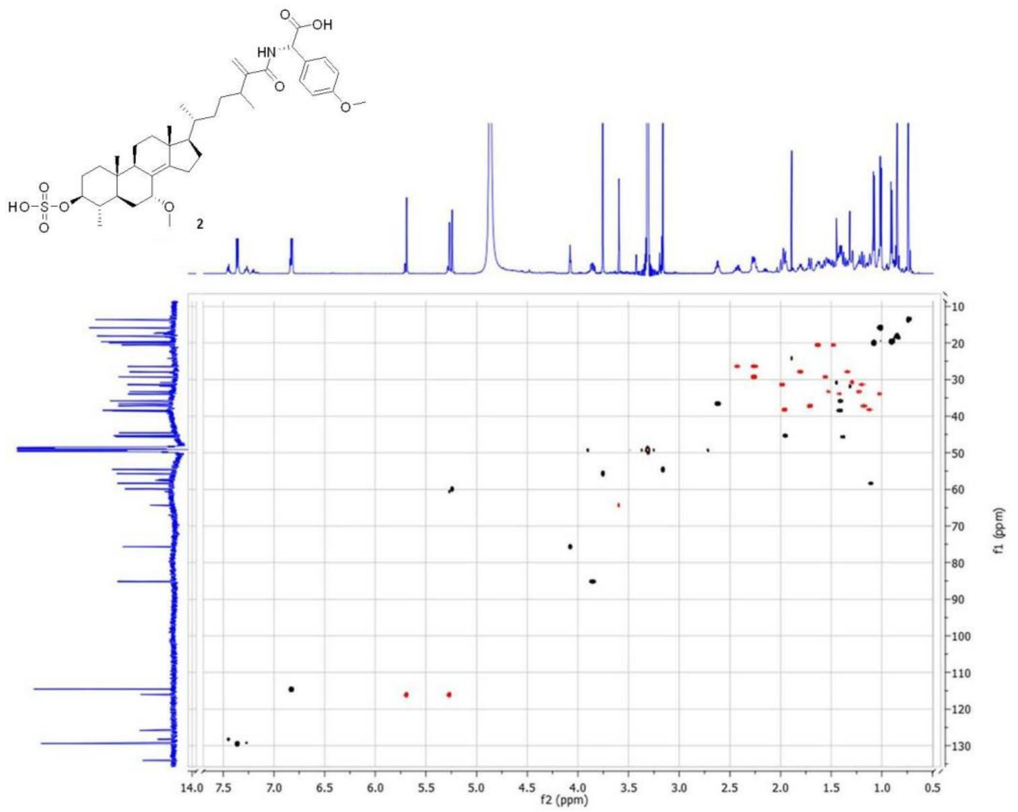

Figure S21. HSQC spectrum (CD_3OD) of compound **2**.

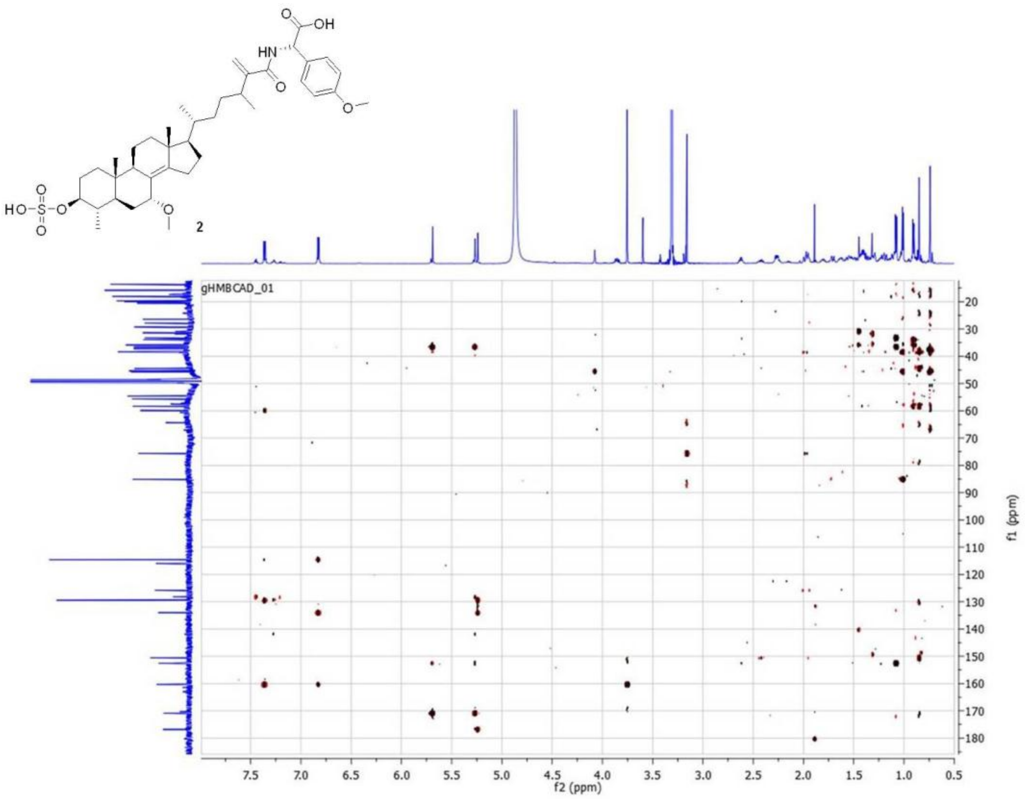

Figure S22. HMBC spectrum (CD_3OD) of compound **2**.

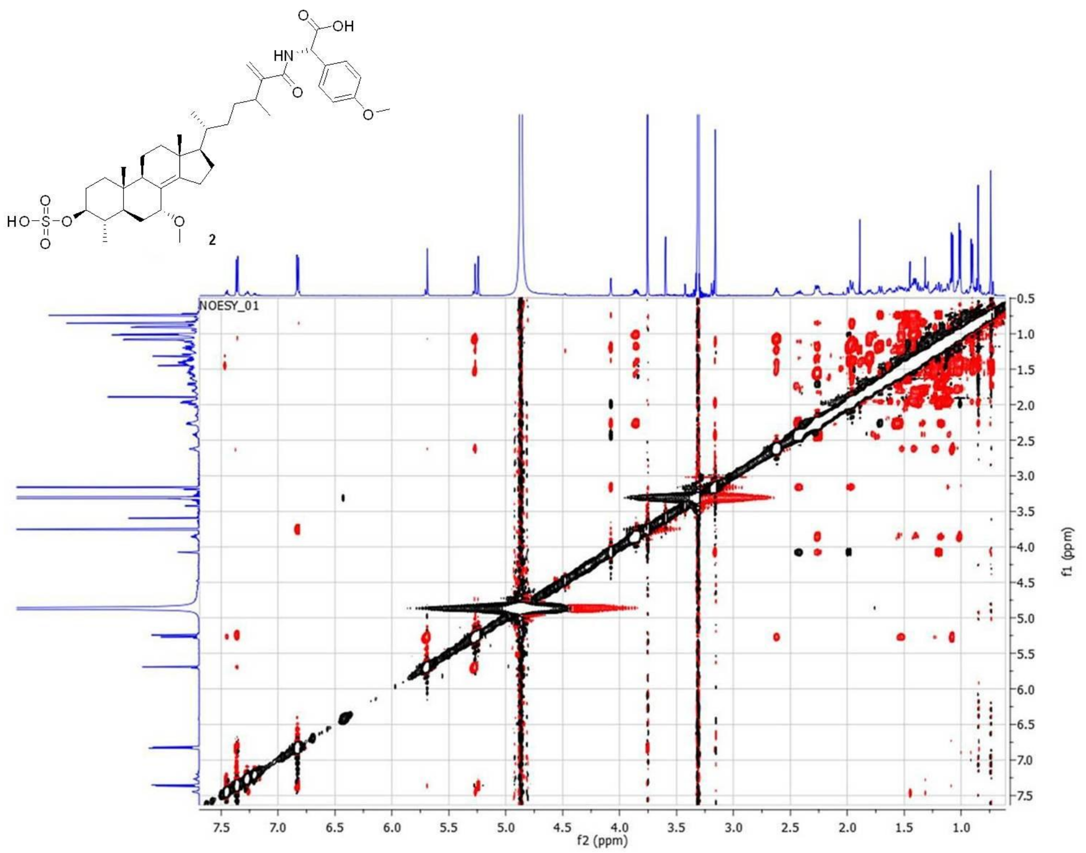

Figure S23. NOESY spectrum (CD_3OD) of compound **2**.

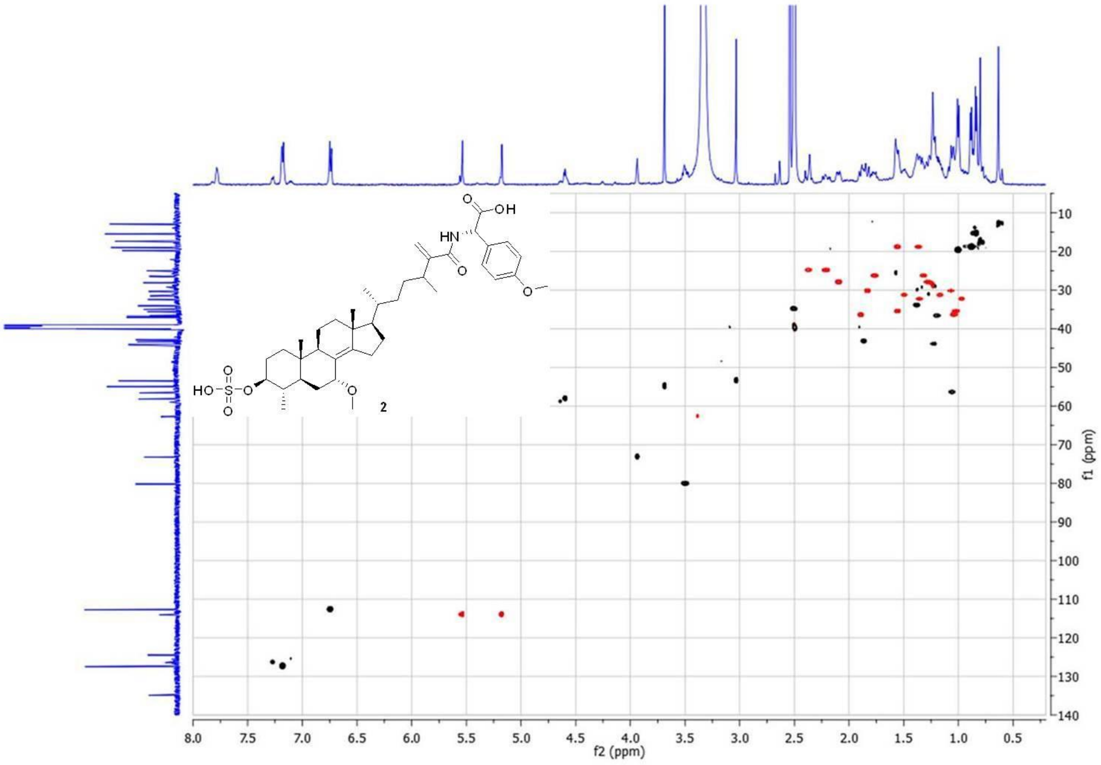

Figure S24. HSQC spectrum (DMSO-d_6) of compound **2**.

Qualitative Compound Report

**Data File**	Poly2neg_2014-07-01.d	**Sample Name**	Poly2
**Sample Type**	Sample	**Position**	P1-A8
**Instrument Name**	Instrument 1	**User Name**	
**Acq Method**	VsmyNegPoly.m	**Acquired Time**	01/07/2014 17:22:37
**IRM Calibration Status**	Success	**DA Method**	Default.m
**Comment**			

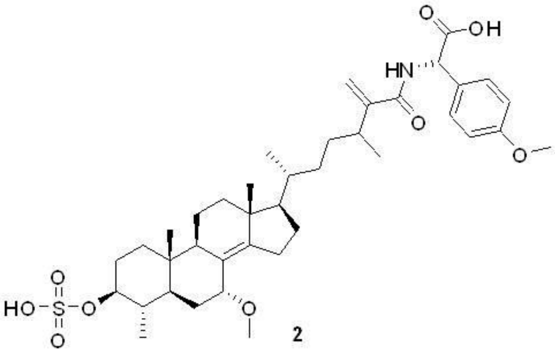

2

Sample Group **Info.**
Acquisition SW 6200 series TOF/6500 series
Version Q-TOF B.05.01 (B5125)

Compound Table

Compound Label	RT	Mass	Abund	Formula	Tgt Mass	Diff (ppm)	MFG Formula	DB Formula
Cpd 1: C39 H57 N O9 S	9.436	715.3749	675238	C39 H57 N O9 S	715.3754	-0.68	C39 H57 N O9 S	C39 H57 N O9 S

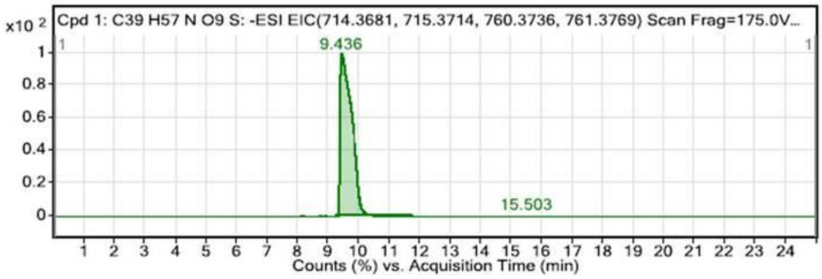

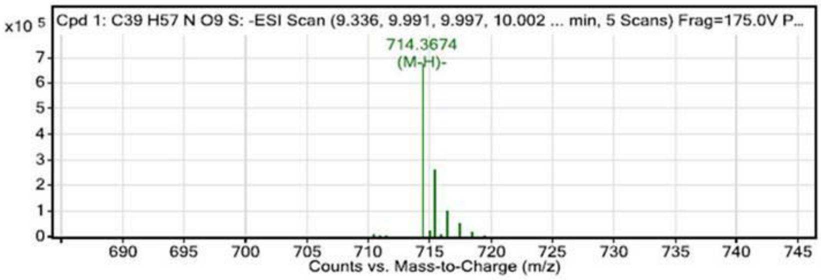

MS Spectrum Peak List

m/z	*Calc m/z*	Diff(ppm)	z	Abund	Ion
714.3674	714.3681	1.07	-1	675238.25	(M-H)-
715.3715	715.3714	-0.08	-1	261685.7	(M-H)-
716.3714	716.3714	0.06	-1	103016.8	(M-H)-

Figure S25. ESIMS spectrum of compound **2**.

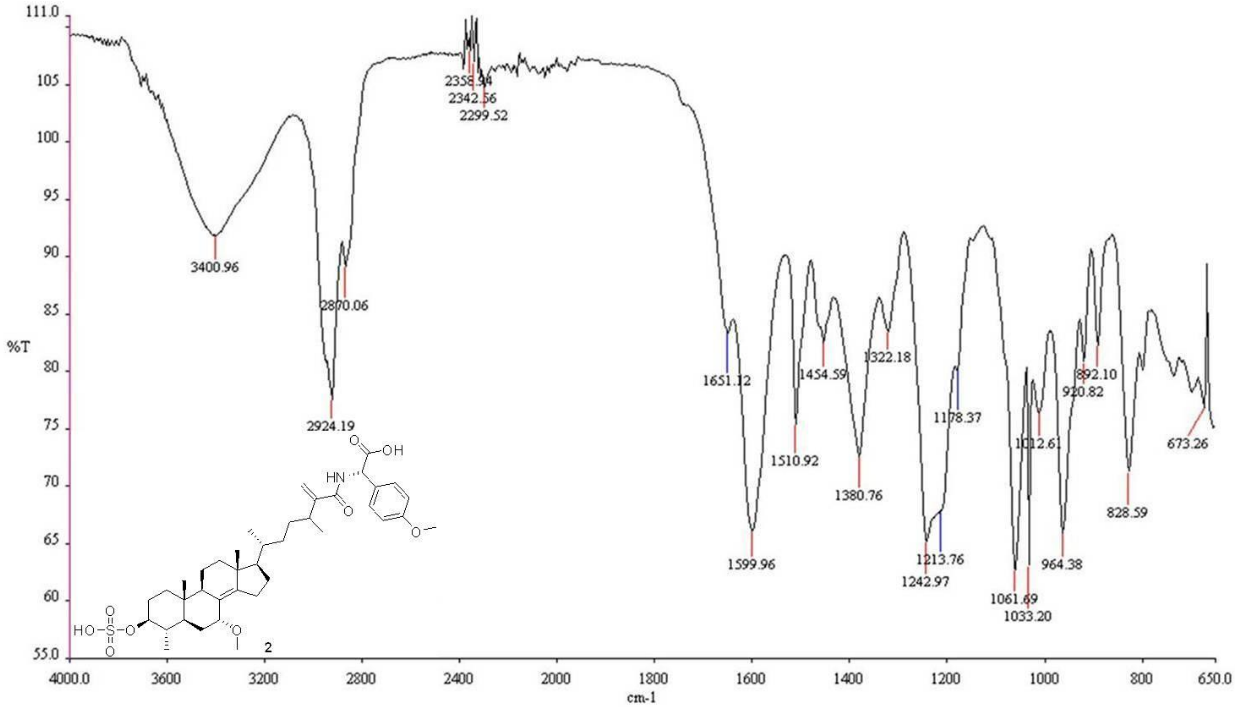

Figure S26. IR spectrum of compound **2**.

© 2015 by the authors; licensee MDPI, Basel, Switzerland. This article is an open access article distributed under the terms and conditions of the Creative Commons Attribution license (http://creativecommons.org/licenses/by/4.0/).
